# Increased sediment load during a large-scale dam removal changes nearshore subtidal communities

**DOI:** 10.1371/journal.pone.0187742

**Published:** 2017-12-08

**Authors:** Stephen P. Rubin, Ian M. Miller, Melissa M. Foley, Helen D. Berry, Jeffrey J. Duda, Benjamin Hudson, Nancy E. Elder, Matthew M. Beirne, Jonathan A. Warrick, Michael L. McHenry, Andrew W. Stevens, Emily F. Eidam, Andrea S. Ogston, Guy Gelfenbaum, Rob Pedersen

**Affiliations:** 1 U.S. Geological Survey, Western Fisheries Research Center, Seattle, Washington, United States of America; 2 Washington Sea Grant, Olympic Peninsula Field Office, Port Angeles, Washington, United States of America; 3 U.S. Geological Survey, Pacific Coastal and Marine Science Center, Santa Cruz, California, United States of America; 4 Washington Department of Natural Resources, Olympia, Washington, United States of America; 5 Polar Science Center, Applied Physics Laboratory, University of Washington, Seattle, Washington, United States of America; 6 Lower Elwha Klallam Tribe, Port Angeles, Washington, United States of America; 7 Univeristy of Washington, School of Oceanography, Seattle, Washington, United States of America; 8 Environmental Protection Agency, Regional 10, Seattle, Washington, United States of America; Northwest Fisheries Science Center, UNITED STATES

## Abstract

The coastal marine ecosystem near the Elwha River was altered by a massive sediment influx—over 10 million tonnes—during the staged three-year removal of two hydropower dams. We used time series of bathymetry, substrate grain size, remotely sensed turbidity, scuba dive surveys, and towed video observations collected before and during dam removal to assess responses of the nearshore subtidal community (3 m to 17 m depth). Biological changes were primarily driven by sediment deposition and elevated suspended sediment concentrations. Macroalgae, predominantly kelp and foliose red algae, were abundant before dam removal with combined cover levels greater than 50%. Where persistent sediment deposits formed, macroalgae decreased greatly or were eliminated. In areas lacking deposition, macroalgae cover decreased inversely to suspended sediment concentration, suggesting impacts from light reduction or scour. Densities of most invertebrate and fish taxa decreased in areas with persistent sediment deposition; however, bivalve densities increased where mud deposited over sand, and flatfish and Pacific sand lance densities increased where sand deposited over gravel. In areas without sediment deposition, most invertebrate and fish taxa were unaffected by increased suspended sediment or the loss of algae cover associated with it; however, densities of tubeworms and flatfish, and primary cover of sessile invertebrates increased suggesting benefits of increased particulate matter or relaxed competition with macroalgae for space. As dam removal neared completion, we saw evidence of macroalgal recovery that likely owed to water column clearing, indicating that long-term recovery from dam removal effects may be starting. Our results are relevant to future dam removal projects in coastal areas and more generally to understanding effects of increased sedimentation on nearshore subtidal benthic communities.

## 1. Introduction

Benthic marine habitats are often defined by their substrate characteristics, yet sediment dynamics such as erosion, transport, and deposition are also important structuring mechanisms in marine ecological communities [1–3]. Human landscape alterations have changed the flux of sediment to the global coastal ocean [4], patterns of deposition and erosion in the marine environment [5], and sediment linkages between catchments and marine environments [6]. For example, activities such as deforestation [7], mining [8], and urbanization [9] have increased the delivery of sediment to coastal habitats, and consequently driven community change in coral reefs [10], seagrass meadows [11], estuaries [12], and rocky reefs [2, 13]. Increased sediment delivery can directly affect habitats and organisms and lead to community-scale changes by burying substrates [14–16], preventing propagules from settling [17, 18], or reducing growth and survival [19, 20]. Sedimentation can also indirectly affect community composition by altering rates of competition and predation, for example, through creating refugia for deposition tolerant species [21, 22].

The scale and magnitude of sediment effects on marine benthos are habitat dependent [1, 23]. Rocky reefs are particularly susceptible to sediment dynamics—particularly deposition—because the presence of hard substrate is required for settlement, attachment, and survival for many species [1]. Community composition in soft sediments can also be negatively affected by sediment deposition [23], particularly if the grain size of the deposited sediment is finer than the original matrix (e.g., a change from coarse sand to mud) [12, 19]. In general, diversity tends to be lower at sites affected by sediment deposition [16, 24–27], but sediment disturbance can increase regional diversity by creating a mosaic of habitat patches in varying states of succession [13].

Increased turbidity in the water column can also affect community composition. The distribution of primary producers, particularly across depth gradients, is reduced by increasingly turbid conditions [2, 28]. Invertebrate communities can also be negatively affected by increased turbidity levels [29], which has been particularly well studied on coral reefs [30]. Filter feeders may benefit when increased turbidity is accompanied by increased particulate organic matter availability [31–33]. Sediment dynamics are thus an important structuring agent for subtidal communities, and can include deposition, substrate fining, and turbidity fluctuations.

Dam removals are becoming more common, particularly in North America [34, 35] but also in other parts of the world (e.g., Europe and Asia) [36–39]. Sediment released during and following dam removal, especially in cases with significant storage of reservoir sediment, is a primary driver of physical and ecological change in fluvial systems [40–42]. In areas near the ocean, dam removal can initiate rapid sediment flux to coastal systems, but in the long run it can restore sediment-habitat interactions and formation processes that were lost when the dams were built. Few published studies exist documenting the effects of dam removal on marine nearshore habitats [38, 43–46], making evaluation of effects in estuarine and marine systems novel and valuable, especially as dam removals begin to outpace large dam construction in the United States [47].

Two dams constructed near the coast on the Elwha River, Washington State, USA, in the early 1900s trapped approximately 30 million tonnes (Mt) of sediment by 2011 [48]. The large sediment volumes released into the river and transported to marine waters after the initiation of dam removal in September 2011 allowed us to examine the physical and biological responses to extremely high sediment fluxes. Using data collected before and during dam removal, we addressed the following questions about the effects of dam removal and associated sediment fluxes on nearshore subtidal biological communities. First, were community changes greater near the river-mouth sediment source than farther away, and if so, what was the nature of the response gradient? Second, were community changes related to physical changes due to increased sediment input, and if so, how? We focused our analyses on community responses to two physical changes: (1) deposition of sand or mud on the seafloor, and (2) increased water column turbidity. Based on hydrodynamic model simulations [45, 49] and Elwha River plume observations [50] we expected increased turbidity to have a larger spatial area of impact than sediment deposition, facilitating our ability to independently test the effects of these physical changes on biological communities. We observed fundamentally different responses among vegetation, macroinvertebrates, and fish, and therefore we present results separately for each of these components.

## 2. Study area and background

**Fig 1:**
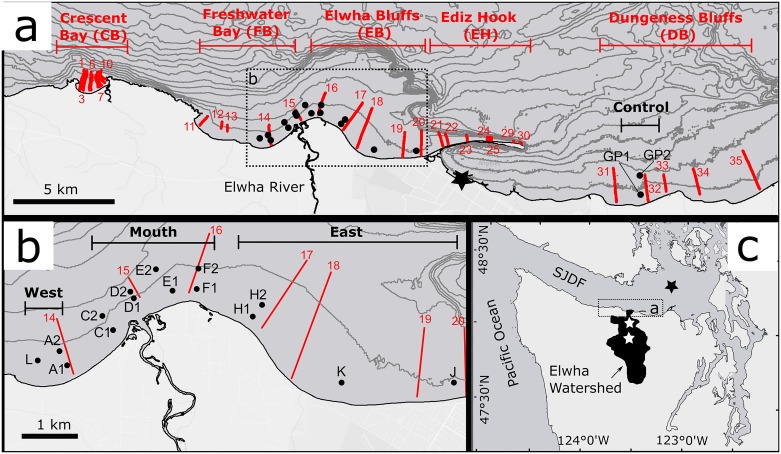
Overview map of the study area on the Olympic Peninsula in Washington State, USA, and locations and names of 17 dive sites (black dots) and 27 towed video transects (red lines) and their associated transect numbers. Five grouping “areas” (Crescent Bay, Freshwater Bay, Elwha Bluffs, Ediz Hook, Dungeness Bluffs) used in the analysis of towed video data are shown in red in panel ‘a’, and four site-groups (Control, East, Mouth, West) used in the analysis of dive site data are shown in black in panels ‘a’ and ‘b’. Abbreviations in parentheses for areas and site-groups are used in tables and figures. The locations of the two former dam sites are shown in panel ‘c’ with white stars. NOAA water level station 9444090 is shown in panel ‘a’, and NOAA wave buoy 46088 is shown in panel ‘c’, both marked with black stars. Ten meter contour lines in panels ‘a’ and ‘b’ are derived from pre-dam removal bathymetry [52–55]. In panel ‘c’, SJDF = Strait of Juan de Fuca.

The Elwha River (Fig 1) originates in a mountainous landscape and descends from an elevation of approximately 1100 m to sea level over 72 km. The Elwha River has a mean annual discharge of 43 m^3^·s^-1^, with a bimodal hydrograph shaped primarily by rain in the winter months and snowmelt in the spring. The discharge magnitude for 2-year recurrence flood is 400 m^3^·s^-1^ [51].

The Elwha Delta protrudes into the Strait of Juan de Fuca, an east-west oriented channel (18–27 km width) connecting Puget Sound and the Strait of Georgia to the Pacific Ocean (Fig 1c). The subaerial delta is perched on a relict delta that forms a broad, shallow shelf extending from the intertidal to a depth of 40–60 m before steeply descending into the main basin of the Strait of Juan de Fuca (Fig 1a). The central Strait of Juan de Fuca is subject to swell propagating from the Pacific Ocean and locally generated wind waves, with a median wave height of 0.4 m [56]. The central Strait of Juan de Fuca experiences mixed semidiurnal tides, with a mean daily range of 1.4 m and a great diurnal range (i.e., the difference between mean higher high water and mean lower low water) of 2.1 m [57].

Flow and current regimes in the Strait of Juan de Fuca play a role in structuring the marine ecosystem. Tidally generated currents can exceed 1 m·s^-1^ [50], resulting in a generally well-mixed and weakly stratified water column [58]. Analysis of residual currents suggests that the Strait of Juan de Fuca is dominated by estuarine flow, with landward movement of bottom water and seaward movement of surface water [58]. Under coastal downwelling conditions, however, a second mode of residual flow is associated with eastward transport of surface water into the Strait of Juan de Fuca. Under these circumstances water masses advected into the Strait of Juan de Fuca from the continental shelf influence surface temperature, salinity, oxygen, and pH [58, 59].

### 2.1 Nearshore habitats prior to dam removal

Prior to dam removal, mixed sand and gravel substrate characterized the nearshore habitat immediately offshore from and east of the river mouth, with increasing abundance of boulders to the west of the river mouth [56, 60]. Rubin et al. [60] distinguished four predominant habitat types in the coastal system adjacent to the Elwha River mouth: bedrock/boulder reef, low relief sand and gravel substrate, moderate relief sand and gravel substrate, and low relief sand substrate. Across all four habitat types, mean density (*N*·m^-2^) was 3.1 for kelp (10 species), 2.7 for invertebrates (65 taxa), and 0.2 for fish (24 taxa). Seavey and Ging [61] used SCUBA surveys to record 57 invertebrate species and 40 macroalgal species, with macroalgal cover exceeding 50% at most sites. Floating kelp canopy (primarily *Nereocystis leutkeana*), assessed with annually aerial surveys since 1989, persisted longer than 15 years at many locations adjacent to the Elwha River delta, particularly central Freshwater Bay [60, 62].

Building the Elwha River dams led the majority of the river’s annual sediment load to be sequestered in the reservoirs, which may have increased the abundance of coarse-substrate habitats near the Elwha delta. However, hydrodynamic modeling and measured current velocities suggest that near-bed shear stress in the nearshore, driven primarily by tidally generated currents, frequently exceeded the critical shear stress for sand [43, 45]. Thus, the relative abundance of gravel/cobble substrates prior to dam removal may also have been controlled wholly or in part by hydrodynamics. A hydrodynamic model by Gelfenbaum and Stevens [49] predicted that dam removal would result in elevated turbidity across the nearshore zone adjacent to the Elwha River delta (Fig 1a), with deposition most likely occurring along a narrow band adjacent to the Elwha River mouth in water depths of < 15 m.

### 2.2 Dam removal

The 32-m tall Elwha Dam was completed in 1912 at river kilometer (rkm) 8. The 64-m tall Glines Canyon Dam, constructed in 1927, was located at rkm 21 (Fig 1c). Approximately 30 Mt of sediment accumulated in the two reservoirs behind the dams prior to removal [48]. The staged removal of the dams began in September 2011. Sediment flux increased dramatically from the downstream reservoir, Lake Aldwell, in March 2012 during the deconstruction of the Elwha Dam, which was completed by April 2012. The reservoir upstream of the Glines Canyon Dam, Lake Mills, started spilling sediment past the dam site in October of 2012 during dam deconstruction, and removal was completed by October 2014. During the first two years of dam removal (ending in September 2013), over 10 Mt of sediment were eroded from the two reservoirs [48] with approximately 3.5 Mt accumulating near the river mouth [45]. Turbidity in the river downstream of the dams increased nearly three orders of magnitude above background levels during the same time period [63, 64].

## 3. Methods

We assessed the effects of dam removal on nearshore subtidal communities using a Before-After-Control-Impact (BACI) approach [65]. Ecological data were collected along gradients of expected impact associated with turbidity and sediment deposition east and west of the river mouth, at 3–17 m depths referenced to mean lower low water (MLLW). We also collected data in two control areas where effects of dam removal were expected to be minimal: Green Point, 21 km east of the Elwha River (“Control” and “Dungeness Bluffs”; Fig 1a); and Crescent Bay, 15 km west of the Elwha River (Fig 1a). Our study was conducted on lands managed by the Washington Department of Natural Resources but lacking any other protection status (i.e., they were not in preserves). No specific permissions were required for our study. We did not observe any endangered or protected species during our study.

### 3.1 Dive sites

We evaluated biological and physical habitat changes before and after dam removal at 15 impact and two control sites (Fig 1) using SCUBA (hereafter referred to as “dive sites”). Twelve dive sites in the impact area (A1-H2, Fig 1b), along with the two control sites at Green Point (GP1 and GP2), were established and surveyed prior to dam removal between 2009 and 2011 and surveyed annually through 2014 (S1 Fig). During surveys in 2012, one year after dam removal began, it became clear that dam removal effects on macroalgae extended farther from the river mouth than the previously established impact sites. To better capture the spatial extent of dam removal effects, in 2012 we established three additional impact sites (J, K and L; Fig 1b) at locations where macroalgae had been abundant during reconnaissance surveys made in 2008 [60] and were still at least moderately abundant when resurveyed in 2012. All surveys were conducted during summer between 21 July and 12 September (S1 Table).

**Fig 2:**
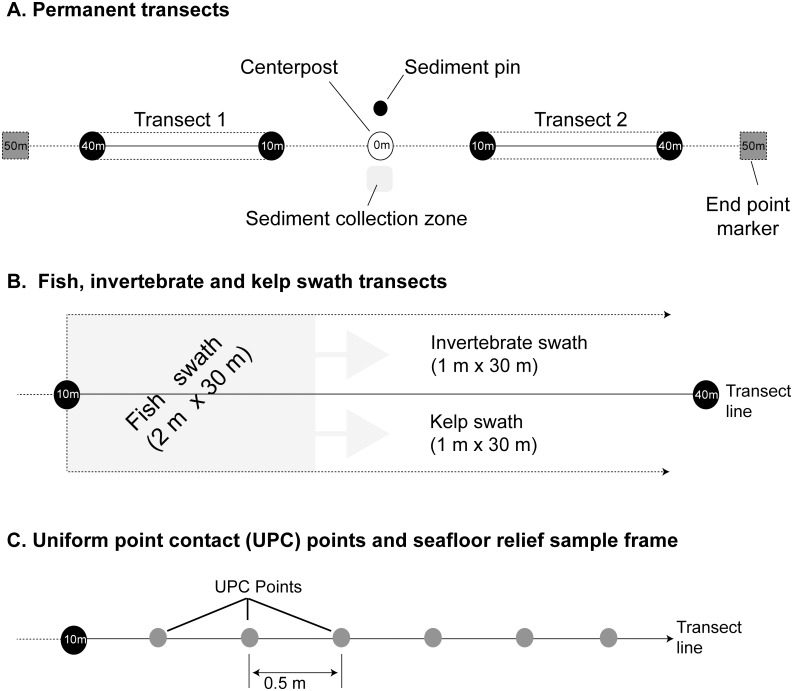
Site schematic for dive sites (A) and methodology for (B) invertebrate, kelp and fish swaths and (C) uniform point contact (UPC) surveys. The two transects at each site are oriented in the alongshore direction.

Dive sites were marked with a stainless-steel post at the center and concrete pier blocks 50 m to the east and west (Fig 2A). Transects were established between the center post and end markers at each site in approximately shore-parallel directions. Surveys were conducted between the 10 m and 40 m points on a measuring tape arrayed between the center post and endpoint marker, with the distal 0–10 m and 40–50 m sections providing buffers to minimize marker-related disturbance.

#### 3.1.1 Biological change at dive sites

We collected benthic community data at each dive site using methods adapted from the Partnership for Interdisciplinary Studies of Coastal Oceans (PISCO) kelp forest monitoring program [60, 66]. Individual organisms present in a 1-m wide swath along the 30-m transect (Fig 2B) and exceeding a size threshold (≥ 2.5 cm in any dimension for invertebrates, and > 24 cm long from holdfast to blade tip for kelp [Laminariales] and acid kelp [Desmarestia]) were identified to the lowest practicable taxonomic level and tallied. When divers were unable to identify taxa *in situ*, we used underwater photography and taxonomic keys on the surface to resolve unidentified taxa. Species were tallied in 10-m segments within each transect. Abundant species were subsampled by noting the segment length at which 30 individuals were present within a 10-m transect segment. In addition to enumerating taxa as described above, red algae > 24 cm long were classified into four growth forms: branched (flat branching blades), leaf (leafy blade), bushy (cylindrical branches), and lacey (filamentous/dense). Each growth was recorded as present or absent in six 5-m transect segments. We calculated a fleshy red algae presence/absence index for each growth form as the number of segments where the growth form was present divided by six.

We define primary cover as the surface area of substrate occupied by the attachment structures of vegetation or sessile invertebrates. Percent primary cover was estimated using a uniform point contact (UPC) methodology [60]. At points spaced every 0.5 m along each transect (N = 60 points per transect; Fig 2C), we recorded the type of organism attached to the substrate or the substrate type (bare rock or bare sand) if it was unoccupied. The UPC methodology was also used to estimate percent secondary cover of brown, red, green, or drift algae. We define secondary cover of macroalgae as the surface area of substrate having macroalgal foliage laying over the substrate but not attached to it. We assessed the presence or absence of foliage of each type of algae at each UPC point.

Fish were tallied in a 2 m x 2 m moving window as the transect tape was deployed (Fig 2B). Additionally, any fish taxa observed during the invertebrate or kelp surveys that were undetected during the fish survey (typically small, cryptic taxa) were included in fish density estimates.

Finally, each transect was videoed by a diver swimming 1–2 m above the substrate [67]. These videos served as an additional record to corroborate uncertain quantitative estimates, confirm presence/absence data, or otherwise verify *in situ* data.

### 3.2 Towed video transects

We used a towed underwater video system (Splashcam Deep Blue Pro recorded to Digital8 tape) to assess subtidal changes to marine algae and seagrasses over a broader area of the Elwha River delta [68–70]. We identified five geographic areas (Fig 1a) based on geomorphic features and hypothesized effects of reduced sediment input before dam removal [71]. Within each area, five to ten randomly selected transects were run perpendicular to shore to estimate macrovegetation parameters using a line intercept sampling approach. An 11-m shallow-draft vessel traveling at approximately 1 m·s^-1^ collected imagery of a 1-m wide swath of seafloor along each transect. Spatial positions were logged simultaneously with a differential GPS (Trimble AgGPS 132) and water depth was measured with an echosounder (Biosonics DE 4000) [69]. Echosounder depth was adjusted to local MLLW based on observed water levels at NOAA tide station 9444090 in Port Angeles (Fig 1a). Each transect was surveyed to a maximum depth of approximately 15 m (MLLW), the deep extent of abundant macrovegetation beds determined from reconnaissance surveys. The minimum depth of transects extended to either the shallowest navigable water depth (about 2 m) or the edge of floating kelp beds (which preclude towed imagery collection due to entanglement). Surveys were conducted in 2010 and annually during 2012–2014 between 8 July and 31 August on days when water clarity was sufficient for high quality video (S2 Table).

During post processing, a frame from every fifth second of video imagery was classified using a modified Braun-Blanquet [72] scale into five categories of percent cover: < 15%, 15–33%, 34–66%, 67–84%, and > 85%. Vegetation > 10 cm in size was visually identified using a distance-calibrated set of lasers for scale. Over 27,000 images were classified during the four survey years.

Five categories of vegetation were visually identified: all macrovegetation, kelp in the taxonomic order Laminariales, seagrass species in the family Zosteraceae, green algae, and other red/brown algae. Vegetation categories were generally easy to distinguish based on color and morphology, except the diminutive kelp *Laminaria ephemera* and ligulate species in the non-kelp genus *Desmarestia*. These taxa were classified according to the species composition of voucher samples collected with a Van Veen grab in areas where they co-occurred.

### 3.3 Evaluating sediment change

We evaluated ecological changes driven by sediment fluxes and deposition by quantifying deposition and grain size change at dive sites, as well as water column turbidity at dive site and towed video transect locations.

#### 3.3.1 Substrate change

In analyses of biological response at dive sites we treated substrate change as a categorical variable (changed or unchanged). We defined substrate change as bed elevation increase ≥ 10 cm and (or) UPC percent sand increase ≥ 30% (e.g., from 20% to 50%; see UPC grain size classifications below) relative to measurements made before dam removal.

We evaluated bed elevation change at each dive site using one of two methods. First, at each dive site we pounded a 1 m piece of rebar into the substrate until refusal (typically about 60 cm). We measured (± 2 cm) the distance from the top of the pin to the bed during every site visit. We also estimated bed elevation (± 13 cm) from digital elevation models (DEM) created during bi-annual topography and bathymetry surveys of the Elwha delta [45, 73]. Measurements from DEM were used to assess bed elevation change only at sites where the sediment pin was buried, lost, or dislodged from the substrate.

Water depth at each dive site before dam removal was used as a covariate in some of our biological analyses. We used water depth estimated from dive computer pressure sensors (rather than from DEM because some sites were beyond the DEM spatial extent) adjusted to MLLW using tide height logged at NOAA Station 9444090 (Fig 1a).

We evaluated substrate grain size change at dive sites using two methods. First, we collected a large (typically 1–2 kg) sediment sample at each site using a 15-cm diameter tube pushed 15 cm into the substrate or to refusal. Each sample was allowed to settle for > 12 hours, decanted to remove as much water as possible, transferred to a sample bag and frozen. All samples were analyzed for grain size distributions at the USGS Pacific Coastal and Marine Geology Science Center (PCMSC) Sediment Laboratory (Santa Cruz, California). Samples were homogenized, split, and run through a Coulter counter and sieves, providing grain size distributions from 0.0001 to 16 mm.

We also evaluated substrate grain size at each dive site using the UPC method [60]. At 60 points spaced every 0.5 m along each 30-m transect (Fig 2C) we classified substrate as bedrock, boulder (> 25 cm), cobble (6–25 cm), gravel (0.2–6 cm), or sand (< 0.2 cm).

#### 3.3.2 Water column turbidity

We used MODIS satellite imagery and supporting data from the NASA MODIS Adaptive Processing System (MODAPS) as a proxy for water column turbidity at dive sites and along towed video transects [74]. Using this proxy was the only way for us to have comparable turbidity data across our study area for the duration of the study. For our analysis we included the MODIS Aqua MYD02QKM Level 1B Calibrated, Geolocated Radiance/Reflectance Data Set (MYD02, 250 m nominal resolution), the MYD03 Geolocation Data Set (MYD03) and the MYD35_L2 Cloud Mask Data Set (MOD35, 1000 m nominal resolution) [75].

MYD02 and MYD03 data were processed to produce remote sensing reflectance as described by Hudson et al. [74]. Remote sensing reflectance was then corrected for variations in the solar zenith angle using MYD03 data and was corrected for atmospheric effects via dark object subtraction [76]. Only pixels classified as “confident clear” by MYD03 were included and all images were screened manually for clouds. Individual data sets for the period from 2008 to 2014 were stacked, and time-series extracted for dive site and video transect segment locations (division of each video transect into multiple segments is described in 3.5.1 below).

We summarized reflectance each year as the 90^th^ percentile of measurements recorded at various time points during the water year (October-September; S3 Table). Reflectance data for water years 2008–2011 were pooled to characterize 90^th^ percentile reflectance before dam removal. Reflectance sample size (i.e., number of clear days) was 129 before dam removal and 24–33 in years after the start of dam removal (S3 Table). For analyses, 90^th^ percentile reflectance for a water year was matched with the biological survey near the end of that water year.

We used the 90^th^ percentile rather than the median in part because during dam removal, reflectance sample size tended to be higher in months when mean reflectance was low (S3 Table); using the 90^th^ percentile compensated for overrepresentation of low reflectance values in the data set. We also examined within-year spatial correlations between reflectance and brown algal density at dive sites (i.e., data were reflectance and density at each site, *N* = 17) and found stronger correlations for 90^th^ percentile reflectance than for the median. Similarly, we found stronger within-year correlations between reflectance and brown algal density for water year 90^th^ percentile reflectance than for growing season (March-August) 90^th^ percentile or median reflectance.

### 3.4 Environmental background conditions

Magirl et al. [64] measured sediment load and river discharge data during the study period at approximately rkm 5 at USGS water-quality station 12046260.

We measured temperature during the study period at Site E1 on the bottom (Fig 1b; depth = 6 m). A Seabird 26+ bursting pressure sensor with a thermistor was deployed on 29 January 2009, recovered and re-deployed at roughly 6 month intervals, and recovered for the final time on 13 March 2011. Temperature observations were logged hourly. Temperature observations were extended beyond September 2011 using an Onset HOBO temperature and light sensor that was recovered and swapped with a new one at 6–12 month intervals; temperatures were logged every 20 minutes.

Wave parameters were measured at sites near the Elwha River delta [56, 77, 78] but not continuously. To assess the temporal variability in waves influencing the Elwha River delta we used hourly significant wave height and dominant period estimates from the Hein Bank Buoy [79] in the central Strait of Juan de Fuca (Fig 1c). Wave energy flux was derived from reported significant wave height and dominant wave period using common analytical expressions [80] and averaged by day.

### 3.5 Analysis

We used several types of analyses on the dive and towed video data sets to address three main questions of concern for this paper. The questions and analysis types are summarized in Table 1. Below we describe the major features of the analyses to give readers an understanding of our approaches and why we used them. Further technical details of the analyses are given in S1 Text.

**Table 1:** Summary of statistical analyses performed on dive and video data to address three questions.

Analysis	Dataset	Predictors		Uni/Multi	Response	Type
		Fixed	Random			
***Question 1*: *Was biological change after dam removal greater near the river mouth than farther away*?**						
Repeated measures (BACI)	Dive	SiGr, Yr, SiGr*Yr	Site(SiGr)	Univariate	Per metric	Linear^a^^,^^b^
				Multivariate	Community	Linear^c^
	Video	Area, Yr, Area*Yr	Seg(Trans(Area))	Univariate	Per taxon	Linear^a^^,^^b^
***Question 2*: *Was biological change related to increased reflectance and (or) substrate change*?**						
Multiple regression	Dive	IR (or IA), ID, IS, SC, CR(or CA, Yr)	None	Univariate	Per metric	Linear^d^
					Macroalgae cover	GAM^e^
				Multivariate	Community	Linear^f^
	Video	CR, ID	Seg(Trans)	Univariate	Per taxon	Linear^g^
					Vegetation cover	GAMM^h^
***Question 3*: *Did biological response differ between sediment deposition scenarios (sand on gravel versus mud on sand)*?**						
Repeated measures	Dive	IB, Dep, IB*Dep	Site(IB)	Univariate	Per metric	Linear^a^^,^^b^
				Multivariate	Community	Linear^c^

BACI = Before-After-Control-Impact. SiGr = site-group, Seg = segment of video transect, Trans = video transect, IR = initial reflectance, IA = initial algal cover, CR = change in reflectance, CA = change in algal cover, Yr = year, ID = initial depth, IS = initial percent sand, SC = substrate change (categorical, yes or no), IB = initial substrate (categorical, gravel or sand), Dep = deposition (categorical, before or after). IA and CA were used as alternatives to IR and CR for invertebrate and fish analyses only (see text). Yr was used as an alternative to CR or CA to test for regional drivers (see text). GA(M)M = generalized additive (mixed) model.

^**a**^program *lme4* in R [81];

^**b**^program *phia* in R for post-hoc testing [82];

^**c**^Permanova run in program *permanova* in Primer 7 [83];

^**d**^program *stats* in R [84];

^**e**^gam run in program *mgcv* in R [85];

^**f**^DISTLM run in program *Permanova* in Primer 7 [83];

^**g**^program *nlme* in R [86];

^**h**^gamm run in program *mgcv* in R [85].

#### 3.5.1 Preliminary data treatment

We averaged dive survey data from the two transects at each site to obtain a single data point for each site per year. We then averaged over years before dam removal for sites where data were collected for more than one year (S1 Fig) to obtain a single data point representing initial conditions at each site (hereafter referred to as “Before”). Two data points were missing (site K-Before because K was not surveyed before dam removal and site D1-2014 because D1 became intertidal between the 2013 and 2014 surveys due to sediment deposition); therefore, we used 66 data points for all analyses (S1 Fig).

To assess spatial patterns of vegetation within habitat and depth zones from towed-video surveys, we divided each towed-video transect into contiguous 200-m segments with approximately 30 samples per segment. Transect length varied according to the seabed slope because transects were surveyed to a maximum depth of about 15 m MLLW. At the tip of Ediz Hook, steep bathymetry limited some transects to a single segment of 40 m (Fig 1a). For each segment, mean percent cover was calculated based on the midpoint of the cover category for each classified frame. Mean depth per segment was calculated from echosounder depth. The resulting data set comprised one data point for mean cover and mean depth per segment per year (*N* = 608). Further details of preliminary data treatment are given in S1 Text.

#### 3.5.2 Repeated measures analyses

We used repeated measures ANOVA to test whether biological change during the study period (before and during dam removal) differed between sites near the river mouth and sites farther away (Question 1, Table 1). Dive sites were grouped into four site-groups based on their alongshore location relative to the Elwha River mouth (Fig 1). Video transects were grouped into five geographic areas whose boundaries did not always correspond to the dive site-group boundaries (Fig 1). The repeated measures model included site-group (or area for the video model), year, and their interaction as fixed effects with year treated as a categorical variable. Site nested within site-group was a random effect in the dive survey model. For the video model, the random effect was segment nested within transect nested within area. The repeated measures analysis was a form of BACI analysis because it tested whether before-to-after biological change differed between impact sites near the river mouth and control sites. A finding of significance for the site-group*year interaction term indicated that biological change differed between at least one pair of site-groups. We followed the site-group*year interaction test with pairwise interaction contrasts to determine for which site-group pairs biological change over time differed. We also conducted pairwise simple main effects tests to determine whether biological change differed between pairs of years within each site-group (or area). It should be noted that non-significance of the site-group*year interaction term coupled with significance of the year term indicated temporal change common to all site-groups (i.e., temporal change that was similar between impact and control sites) that could have been due to region-wide effects rather than dam removal. Further details of repeated measures analyses are given in S1 Text.

#### 3.5.3 Multiple regression analyses

We also tested if and how biological responses were related to environmental predictor variables representing spatial and temporal variation in depth, substrate, and sediment input (Question 2, Table 1). Environmental predictors for analyses of dive data were initial reflectance, change in reflectance, initial depth, initial percent sand (from UPC substrate surveys), and substrate change (categorical, yes or no; see 3.3.1. Substrate change). Two additional environmental predictors, initial algal cover and change in algal cover (secondary cover of macroalgae from UPC surveys), were used for analyses of invertebrates and fish only.

Environmental predictors for analyses of video data were initial reflectance, change in reflectance, and initial depth. Information on percent sand and substrate change was not available for most of the towed video study area and therefore could not be included in our analyses. To limit inferences to physical changes associated with dam removal, we excluded the geographic areas where changes in vegetation abundance were small, and any that did occur may not have been due to dam removal (CB and DB; see below). We also excluded segments with mean depth shallower than 6 m (MLLW) to avoid areas near shore where measured reflectance could have included exposed substrate in adjacent intertidal and terrestrial areas. Additionally, we excluded two transect segments near the mouth because at least half of the segment length passed over areas experiencing sediment deposition > 10 cm as determined by the bathymetric surveys, and eight transects because they were so closely located to another transect (i.e., within 100 m) that they corresponded to a single value in the reflectance dataset (250 m resolution). The resulting data set included 212 observations, each representing a transect segment in a year.

Initial values (i.e., Before values) of reflectance, depth, percent sand, and algal cover were measurements made before dam removal. Change in reflectance and algal cover each year after dam removal was calculated as the difference between a given year and the initial value. For each dive site or video transect segment, each year (including Before) was assigned a value for initial reflectance, change in reflectance (always 0 for Before), and initial depth. For each dive site, each year was additionally assigned a value for initial percent sand, substrate change (yes or no; always no for Before), initial algal cover, and change in algal cover (always 0 for Before). Assigning initial values to all years allowed us to separate effects of initial environmental conditions from effects related to dam removal (change in reflectance, substrate, and algal cover). Depth and percent sand at dive sites did not change detectably after dam removal except when/where substrate changed (i.e., from no to yes); thus including substrate change as a predictor accounted for changes in initial depth and initial percent sand.

Collinearity prevented us from including year in the same regression model as reflectance change (or algal cover change) because mean (across sites or transect segments) reflectance change varied among years (as did mean algal cover change). Nevertheless, it was of interest to compare year effects to reflectance change and algal cover change effects. Reflectance and algal cover change were likely due to sediment inputs during dam removal and varied spatially among sites as well as annually. In contrast, yearly changes could be from regional factors unrelated to dam removal, such as recruitment or disease. We therefore compared fit between models that included year and models that included change in reflectance or algal cover. A better fit for models with year may be indicative of regional-scale processes having a stronger effect than dam removal.

We hypothesized that change in macroalgal cover, instead of or in addition to change in reflectance (i.e., turbidity), could affect invertebrates and fish. Collinearity between reflectance change and algae cover change prevented us from including both predictors in the same regression model. We therefore conducted separate multiple regressions with one or the other included. Model fit and predictor effect size indicated whether responses differed between reflectance change and algal cover change. Further details of multiple regression analyses are given in S1 Text.

#### 3.5.4 Analyses of responses to different types of substrate change

We hypothesized that the response to deposition of sand on gravel, which occurred at three of the five dive sites where substrate changed, would be different from the response to deposition of mud on sand which occurred at the other two sites (Question 3, Table 1). We used repeated measures ANOVA to test for response differences between these two types of substrate change. Initial substrate (gravel or sand) and substrate change (no or yes; i.e., before change or after change) were fixed effects; site nested within initial substrate was a random effect. A significant interaction between initial substrate and substrate change would indicate a different response to deposition of sand on gravel from deposition of mud on sand. Sample size for these tests was 19 site-years (5 sites x 4 years—1 site-year [D1-2014]).

## 4. Results

### 4.1 Environmental conditions

During the three years of dam removal, the Elwha River experienced low peak flows (S2A Fig) but very high sediment fluxes. Sediment flux increased immediately following the initiation of dam removal in September 2011, and was elevated through the 3-year period of this study (S2B Fig). Of particular importance was the change in magnitude of sediment flux during the biologically active summer growing season [60]. In the first full year after dam removal started (2012), during the months of April to September, the total sediment flux exceeded 0.65 Mt. During these same months in 2013 and 2014 it exceeded 2.4 Mt and 0.1 Mt, respectively. In comparison, the average total flux for these months for the three years prior to dam removal was around 5000 tonnes.

Wave energy flux at Hein Bank (S2C Fig) was assumed to be a reasonable proxy for wave energy influencing the Elwha River delta. Wave energy flux was seasonal, peaking during the winter months (October to March; S2C Fig). There was little indication of year-to-year variations in wave energy flux during the three years of dam removal.

Subtidal water temperature varied seasonally with a winter median temperature of 8.2°C and a summer median temperature of 9.7°C (S2D Fig). The summer season is generally characterized by greater temperature variability than in winter with oscillations that appear to follow fortnightly spring/neap tidal cycles. During July and October of 2013 two warm water events elevated water temperature to nearly 15°C for multiple days.

### 4.2 Physical change

#### 4.2.1 Substrate change

**Fig 3:**
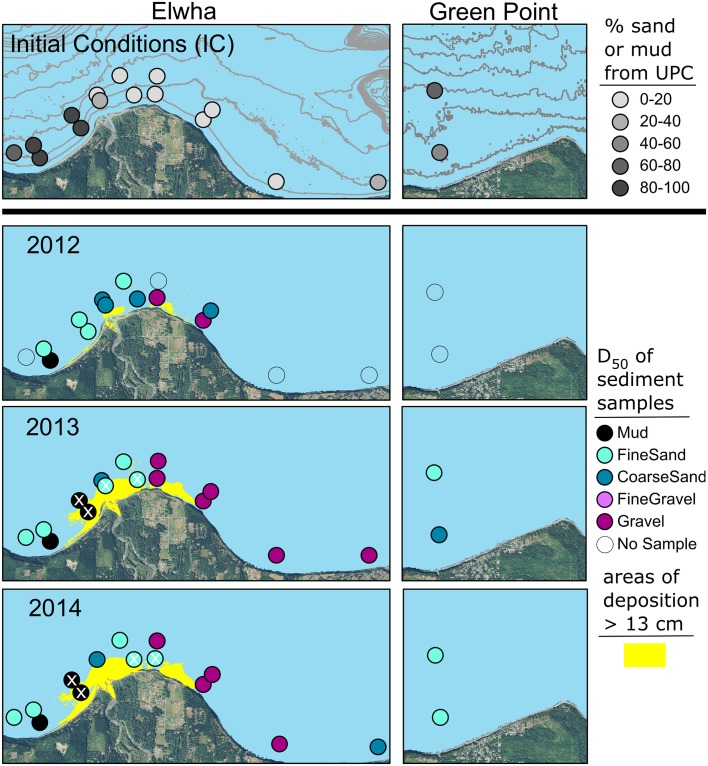
Substrate changes relative to initial conditions before dam removal. Initial conditions are characterized in the top panel by the percent of UPC marks along a 60-m transect identified by divers as sand or mud. The bottom three panels show the median grain size (D_50_) from sediment samples. Also shown are areas of deposition > 13 cm as determined from bathymetric survey data; bathymetric change < 13 cm was considered not detectable due to measurement error [45]. Sites characterized as having experienced substrate change during the study period are marked with a white “X”.

Substrate change data were only collected at dive sites. Persistent alterations to substrate were constrained to five dive sites immediately adjacent to the river mouth (Fig 3; S4 Table). Four of those sites changed due to a combination of sediment deposition and fining of the substrate, whereas at site F1, the substrate fined, but we did not detect a change in bed elevation. Site D1, directly offshore from the river mouth, was buried by 5 m of sediment in 2012, and an additional 8 m of sediment in 2013 at which point it was supratidal and no longer available for sampling. Two formerly sandy sites (C1 and C2) located to the west of the river mouth were buried by ≥ 15 cm of mud between 2012 and 2013, while at site E1 to the east of the river mouth, substrate originally dominated by gravel was buried by 10 cm of sand between 2012 and 2013 (Fig 3; S4 Table).

Substrate at the other 12 dive sites remained comparable to conditions before dam removal. Sites offshore and east of the river mouth, including control sites, were generally dominated by gravel (Fig 3; S4 Table). West of the river mouth, substrate was a combination of sand and mud at sites A1 and A2 and mostly sand with some boulders at site L.

#### 4.2.2 Turbidity

**Fig 4:**
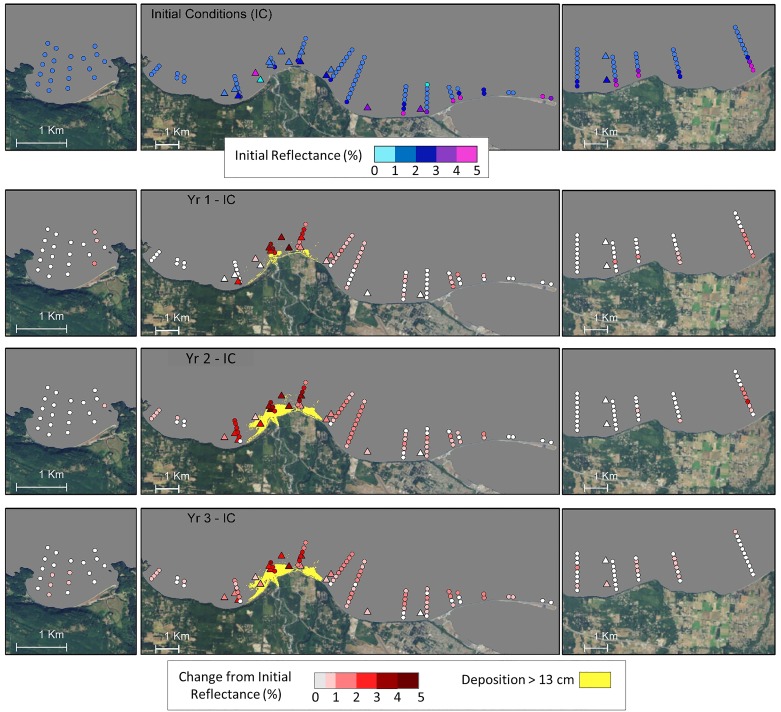
Remote sensing reflectance values for dive sites and video transects for Crescent Bay (left panels), the Elwha Delta area (center panels) and Dungeness Bluffs/Green Point (right panels; Fig 1) derived from MODIS satellite imagery. Values represent the 90^th^ percentile of observations recorded during each water year (October–September). Initial conditions (IC; top panel) for the period before dam removal were averaged for the years 2008–2011 and subsequent panels represent the difference between years 1–3 and initial conditions. Triangles represent dive sites and circles represent video transect segments.

Remote sensing-derived reflectance showed increased surface turbidity at dive sites and towed video transects following the initiation of dam removal (Fig 4). Turbidity increased most within and offshore of the zone of persistent sediment deposition near the river mouth (Fig 4) and remained high in this area throughout the three-year study period. The magnitude of increased turbidity decreased with distance from the river mouth to the east and west, but turbidity was still higher than before dam removal throughout the impact area (Freshwater Bay to Ediz Hook, Fig 1), especially two and three years after initiation of dam removal. Turbidity increased less in the eastern control area (Dungeness Bluffs) than in the impact area. It did not change in the western control area (Crescent Bay).

### 4.3 Vegetation response

#### 4.3.1 Status before dam removal

To set the stage for response to dam removal, we first provide brief summaries of abundance and species composition before dam removal. Total vegetation cover averaged 47% across all towed video transects (S5 Table). At dives sites, total secondary cover derived from UPC, the metric most comparable to total vegetation cover from the video transects, averaged 64%. Brown algae, particularly kelp, and red algae were abundant before dam removal. Mean cover across all towed video transects was 29% for kelp and 19% for other brown algae and red algae combined. Mean secondary cover at dive sites was 39% for brown algae, 17% for red algae, and 7% for combinations of ≥ 2 algal types that were nearly always brown and red algae. Green algae were not abundant before dam removal; mean cover was 1% along video transects and at dive sites. Mean seagrass cover across all video transects before dam removal was 2%, reflecting dense seagrass meadows found at depths less than 10 m in western Crescent and Freshwater bays (transects 1, 2, 3, and 11; Fig 1). Seagrass also grew intermixed with algae in a narrow band along Ediz Hook but was virtually absent from Elwha Bluffs and Dungeness Bluffs (Fig 1). Seagrass did not contribute to secondary cover at any dive site.

We recorded the density of 10 kelp taxa (representing 11 species) and 2 acid kelp taxa (representing 5 species) at dive sites (S6 Table). Before dam removal the most abundant taxa were *Cymathere*, flat-bladed *Desmarestia*, *Pterygophora*, *Saccharina*, and *Alaria*, with total brown algae density averaging 5.7 plants·m^-2^ (S7 Table).

#### 4.3.2 Spatial and temporal changes

**Fig 5:**
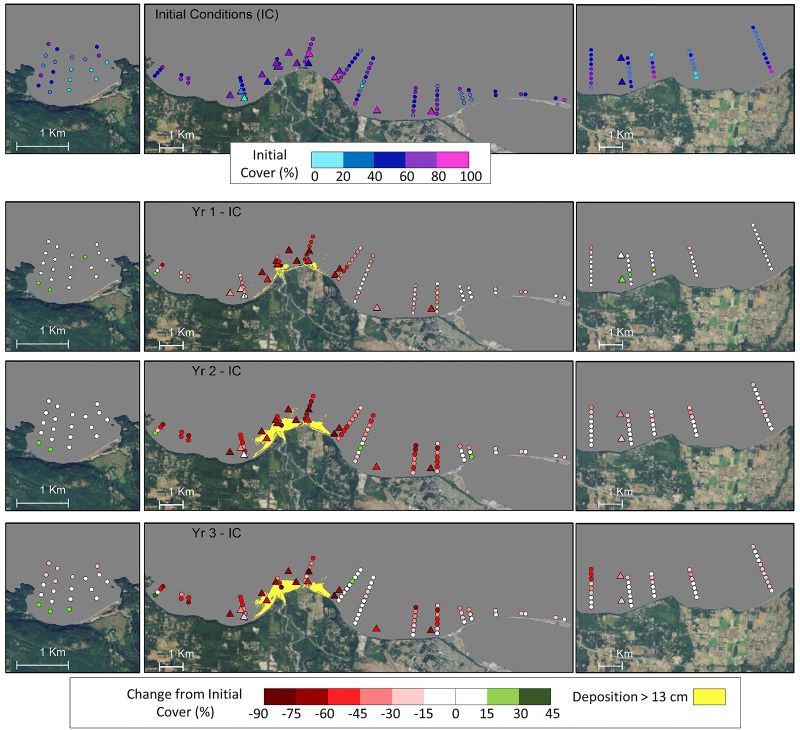
Percent cover of macrovegetation derived from dive surveys (triangles) and video transect segments (circles) for Crescent Bay (left panels), the Elwha Delta area (center panels) and Dungeness Bluffs/Green Point (right panels; Fig 1). Initial conditions (IC) for dive sites are derived from surveys conducted in 2008–2011 before dam removal, and for video transects from video surveys in 2010. Subsequent panels represent the difference between years 1–3 and initial conditions. Triangles represent % secondary cover from UPC surveys at dive sites, and circles represent mean values in the center of 200 m sections of towed video transects.

Combined video and dive data showed a steep decline in vegetation cover near the river mouth in the first year after initiation of dam removal, and intensification and spatial expansion of that decline extending from west Freshwater Bay to the base of Ediz Hook in the second year (Fig 5). Three years after the initiation of dam removal, low algae cover persisted near the mouth except on the east flank of Angeles Point where recovery was observed at video transects 17 and 18 in August 2014 (Fig 5). Algae cover remained low at three nearby dive sites (H1, H2, and K) surveyed a month earlier in July 2014 (Fig 5; see Fig 1 for transect and dive site names/numbers; see S1 and S2 Tables for video and dive survey dates). Vegetation declines in the eastern control area (Dungeness Bluffs/Green Point) were less than in the Freshwater Bay and Elwha Bluffs areas. Vegetation cover was stable at Crescent Bay, the western control area.

**Fig 6:**
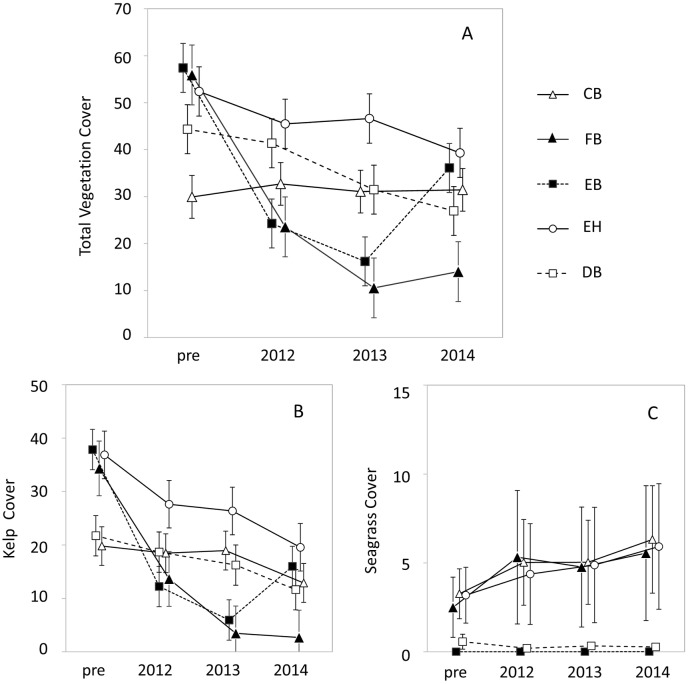
Mean (± SE) percent cover for the towed video areas shown in Fig 1 before (‘pre’) and during dam removal for total vegetation (A), kelp (B) and seagrass (C). Values for total vegetation and kelp are least square means and SEs output from repeated measures ANOVAs. Seagrass values are arithmetic means and SEs. Note that the y-axis scale differs among panels.

Repeated measures analyses for video and dive data confirmed these observed patterns in the spatial extent and temporal sequence of vegetation response. Analysis of video data showed that the pattern of change in vegetation cover over time differed among areas overall and between each pair of areas except EH and DB (Fig 6, Table 2). Extreme declines in total cover occurred one year after dam removal (2012) in the areas bracketing the river mouth (FB and EB), while lower magnitude decline occurred at EH (Fig 6, Table 2). In 2013, cover further declined in FW and EB, and cover decreased significantly in DB relative to conditions before dam removal. In 2014, significant declines were measured in all areas except CB. Some recovery was measured in EB in 2014 which represented an increase relative to 2013 yet a net loss relative to cover before dam removal (Fig 6, Table 2).

**Table 2:** Video data repeated measures (Before-After-Control-Impact) analyses.

Vegeta-tion	Main test *P*-value			Area	Interaction contrast significance level				Within-area significant year pairs (*P* < 0.05)
	Area	Year	Area* Year		CB	FB	EB	EH	
Total	0.115	<0.001	<0.001	CB					None
				FB	***				1–2, 1–3, 1–4, 2–3, 2–4
				EB	***	***			1–2, 1–3, 1–4, 2–3, 2–4, 3–4
				EH	*	***	***		1–2, 1–4, 3–4
				DB	***	***	***	NS	1–3, 1–4, 2–3, 2–4
Kelp	0.249	<0.001	<0.001	CB					1–4, 2–4, 3–4
				FB	***				1–2, 1–3, 1–4, 2–3, 2–4
				EB	***	***			1–2, 1–3, 1–4, 2–3, 3–4
				EH	**	***	***		1–2, 1–3, 1–4, 2–4, 3–4
				DB	NS	***	***	NS	1–3, 1–4, 2–4

**Fig 7:**
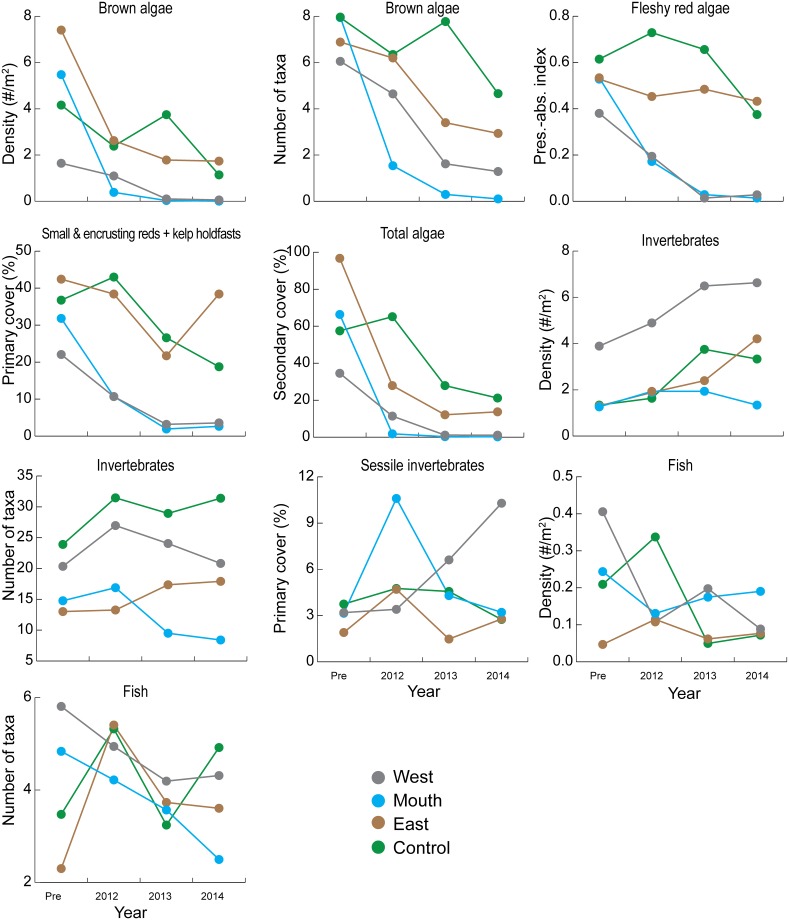
Least-square means before (‘Pre’) and during dam removal from repeated measures tests for univariate algae, invertebrate, and fish responses (see Table 3), for the dive site-groups shown in Fig 1. In cases where values were transformed for the repeated measures tests (see Table 3), displayed means are back-transformed to the original scale.

Areas abbreviations are as shown in Fig 1. The Area*Year interaction term of the main test indicates whether change over time differed between at least one pair of areas. Interaction contrasts indicate for which pairs of areas change over time differed. NS = not significant. Within each area, year pairs with significant change over time are listed; year 1 is before dam removal; years 2–4 follow initiation of dam removal (2012, 2013 and 2014). *P*-values of multiple comparisons are adjusted by the Holm method. Asterisks indicate interaction contrast significance levels:

*** = *P* < 0.001;

** = *P* < 0.01;

* = *P* < 0.05;

Patterns in secondary cover of macroalgae at dive sites were largely consistent with vegetation cover along video transects. Change in total secondary cover over time differed among site-groups overall and between most site-group pairs (Table 3). For Mouth, West, and East, declines in cover were steepest one year after dam removal (2012), and further significant declines occurred between 2012 and 2013 for Mouth and West but not East (Fig 7, Table 3). Unlike the video data, the dive data did not show any recovery in 2014.

**Table 3:** Dive data repeated measures (Before-After-Control-Impact) analyses.

Group	Response	Main test *P*-values			Significant inter-action contrasts (*P* < 0.05)	Within-SiGr significant year pairs (*P* < 0.05)			
		SiGr	Yr	SiGr*Yr		West	Mouth	East	Control
Macroalgae	Brown algae density, assemblage multivariate similarity	<0.001	<0.001	<0.001	W-M, W-E, W-C, M-E, M-C, E-C	None	1–2, 1–3, 1–4	None	None
	Total brown algae density^a^	<0.001	<0.001	<0.001	W-M, W-E, W-C, M-E, M-C	1–3, 1–4, 2–3, 2–4	1–2, 1–3, 1–4, 2–3, 2–4	None	None
	Number of brown algae taxa^a^	<0.001	<0.001	<0.001	W-M, W-C, M-E, M-C	1–3, 1–4, 2–3, 2–4	1–2, 1–3, 1–4, 2–3, 2–4	1–4, 2–4	None
	Fleshy red algae presence/absence index, assemblage multivariate similarity	<0.001	<0.001	0.002	W-E, M-E	None	1–3, 1–4	None	None
	Mean fleshy red algae presence/absence index	<0.001	<0.001	<0.001	M-E, M-C	1–3, 1–4	2–3, 2–4, 3–4	None	2–4
	Primary cover (%) of small fleshy and encrusting reds and kelp holdfasts, assemblage multivariate similarity	0.019	0.001	0.011	M-E	None	1–3, 1–4	None	None
	Total primary cover (%)^a^	0.048	<0.001	0.042	M-E	1–3, 1–4	3–4	None	None
	Secondary cover (%) of algae phyla, assemblage multivariate similarity	<0.001	<0.001	<0.001	W-M, W-E, W-C, M-E, M-C	None	1–2, 1–3, 1–4	None	None
	Total secondary cover (%)^a^	<0.001	<0.001	<0.001	W-M, W-E, W-C, M-E, M-C, E-C	1–2, 1–3, 1–4, 2–3, 2–4	1–2, 1–3, 1–4, 2–3, 2–4	1–2, 1–3, 1–4	None
Invertebrates	Density, assemblage multivariate similarity	0.003	<0.001	0.262	E-C	None	None	None	None
	Total density^a^	0.298	0.130	0.856	W-M, W-E, W-C, M-E, M-C, E-C	None	None	None	None
	Number of taxa^a^	0.112	0.811	0.499	None	None	None	None	None
	Primary cover (%) of sessile and encrusting species, assemblage multivariate similarity	0.040	0.005	0.498	None	None	None	None	None
	Total primary cover (%)^a^	0.601	0.416	0.379	None	None	None	None	None
Fish	Density, assemblage multivariate similarity	0.002	0.001	0.077	None	None	None	None	None
	Total density^a^	0.259	0.463	0.610	None	None	None	None	None
	Number of taxa^a^	0.278	0.236	0.208	None	None	None	None	None

SiGr = site-group (see Fig 1); Yr = year. The SiGr*Yr interaction main test indicates whether change over time differed between at least one pair of site-groups. Interaction contrasts indicate whether change over time differed between each pair of site-groups. Site-group pairs that differed in change over time are listed; W = West, M = Mouth, E = East, C = Control. Within each site-group, year pairs with significant change over time are listed; year 1 is before dam removal; years 2–4 follow initiation of dam removal (2012, 2013 and 2014). *P*-values of multiple comparisons are adjusted by the Holm method.

^a^Ln(y+1) transformed.

Similar to total vegetation, each group of macroalgae declined during dam removal. Kelp cover at video transects responded similarly to total vegetation cover (Fig 6, Table 2). Brown algal density and number of brown algae taxa at dive sites declined similarly to total secondary cover of macroalgae (Fig 7; Table 3). The mean fleshy red algal index and total primary cover showed a somewhat different response: steep declines one and two years after dam removal for Mouth and West sites but less steep declines for East and Control (Fig 7; Table 3). All individual brown algae taxa declined at Mouth sites, with all except *Pterygophora* (a stipitate perennial species) completely absent or nearly so by 2012 (S3 Fig). *Pterygophora* at Mouth sites in 2012 had intact stipes but their blades were often missing or damaged.

**Fig 8:**
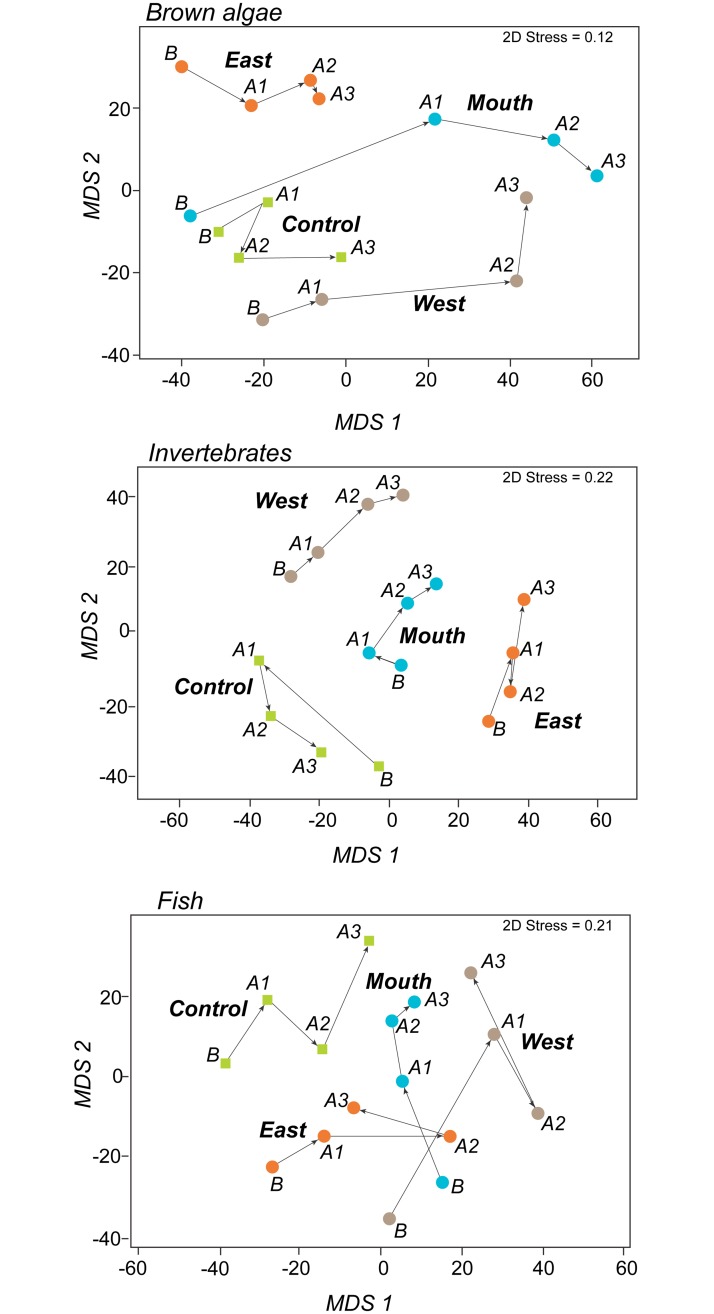
Metric MDS plots showing distance among site-group-by-year centroids for assemblage multivariate similarity. Arrows connect consecutive years (B = before dam removal started, A1-A3 = 1–3 years after the initiation of dam removal) within each site-group (see Fig 1). Axis scales measure Bray-Curtis dissimilarity (for example the dissimilarity between B and A3 for brown algae is 85%) and can be compared between plots (for example brown algae changed more across years than did invertebrates). Time trajectory direction (i.e., arrow direction) indicates direction of community change within plots but is not comparable between plots.

Macroalgal community responses were similar to responses of individual taxa. Change in the brown algal assemblage over time differed among site-groups (Table 3) and was greatest at Mouth sites and least at Control sites (Fig 8). Likewise, change over time for the fleshy red algae, primary cover, and secondary cover assemblages differed among site-groups overall, and between site-groups for at least one of the site-group pairs that included Mouth (Table 3).

The patchy distribution of seagrass on video transects (zero cover at most transect segments versus high cover at a few) invalidated repeated measures analysis due to violations of normality and heterogeneity assumptions. Nevertheless, data on change in seagrass cover after dam removal suggest that seagrass may not have declined as much as macroalgae (Fig 6). Trends in mean seagrass cover in Freshwater Bay were primarily driven by the two segments of transect 11 (Fig 1) closest to shore where seagrass was abundant before dam removal and remained so during dam removal (Fig 5). Similarly, seagrass on Ediz Hook did not decline during dam removal (Fig 6).

#### 4.3.3 Response to substrate and turbidity changes

**Fig 9:**
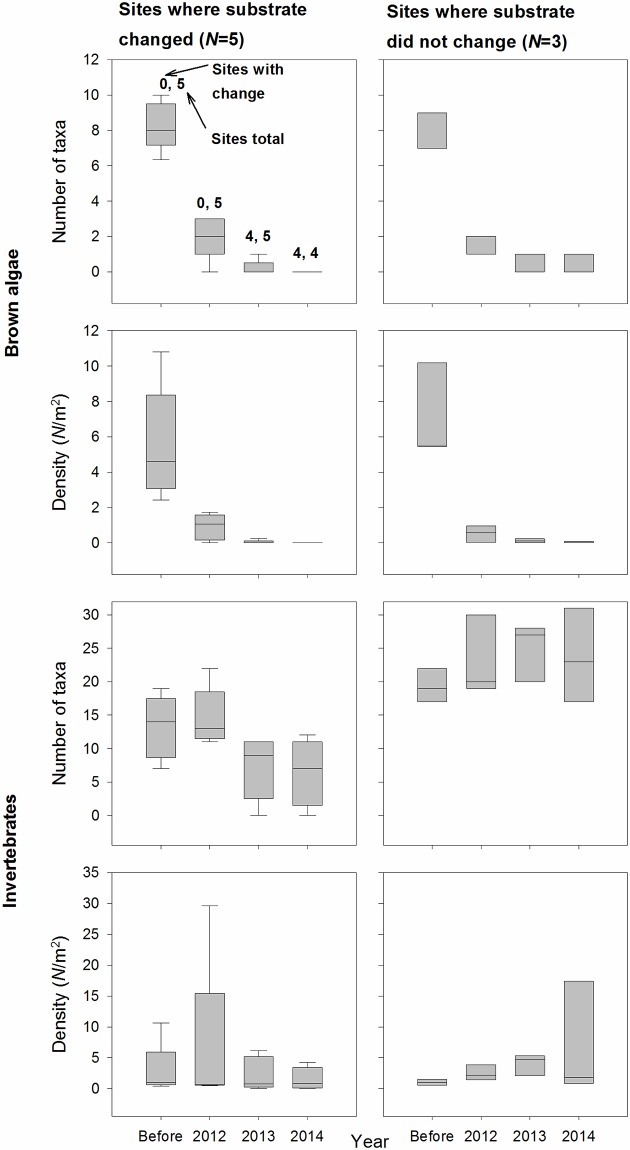
Time series of responses for sites in the Mouth site-group where substrate changed and substrate did not change. Note that one of the sites where substrate changed in 2013 was not sampled in 2014 because it became intertidal due to sediment deposition. Lower whisker = 5th percentile, lower box edge = 25th percentile, line = median, upper box edge = 75th percentile, upper whisker = 95th percentile.

The five dive sites that experienced substrate change were all in the Mouth site-group (Figs 1 and 3). Substrate did not change at any of the Mouth sites in 2012, yet the number of brown algal taxa and total brown algal density decreased at all of them that year (Fig 9). Substrate changed at four Mouth sites in 2013 and one more in 2014, and there was nearly a complete loss of algae associated with the substrate shift. However, there were also algal declines from 2012 to 2013 at three Mouth sites that did not experience substrate change, and no algae rebounded at those sites in 2014 despite the continued absence of substrate change (Fig 9).

**Fig 10:**
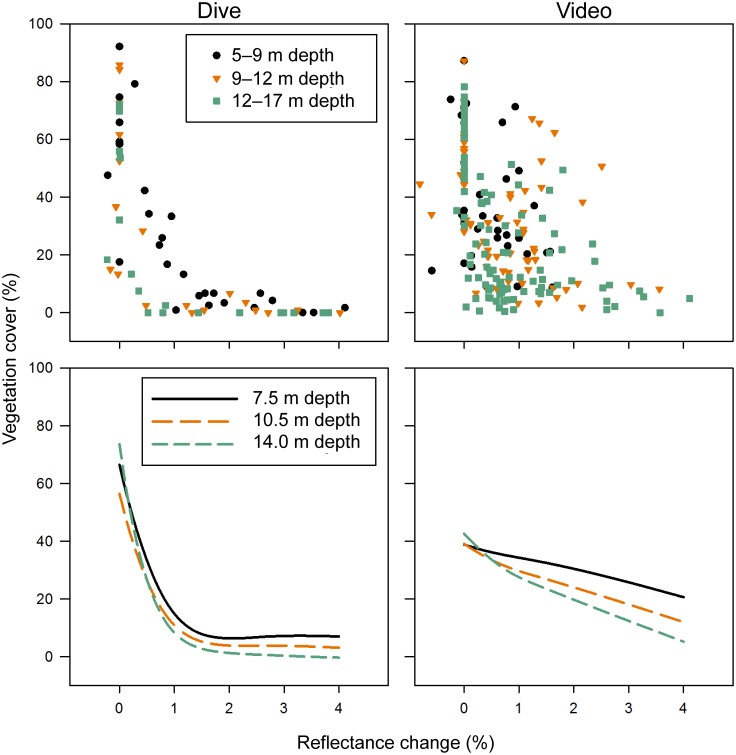
Vegetation cover versus light reflectance change from dive surveys (left) or video transects (right). Vegetation cover is entirely macroalgae for dive surveys but includes a small amount of seagrass for video transects. Top left: one data point per site per year; top right: one data point per transect segment per year; points are color/shape coded by initial depth bin. Curves in the bottom panels are coded by initial depth and were derived from generalized additive models (GAMs). In addition to reflectance change, the dive GAM included initial depth, the interaction between reflectance change and initial depth, initial percent sand, and substrate change (yes or no) as predictors, and the video GAM included initial depth and the interaction between reflectance change and initial depth (S8 Table). The curves were generated by predicting vegetation cover over the range of reflectance change while holding depth constant at each of the three depths shown (and holding the other predictors in the dive GAM constant at their means: 40% initial sand and substrate change = no). Note that the dive GAM was fit to ln(y + 1) transformed vegetation cover; the curves were then back-transformed for display here.

For both dive and video data, there was a nonlinear relationship between vegetation cover and reflectance change (used here as a proxy for water column turbidity change) (Fig 10; S8 Table). With increasing reflectance change, vegetation cover decreased more steeply for reflectance change values between 0 and about 1% than for reflectance change > 1%. Further, the relationship between vegetation cover and reflectance change differed with depth (Fig 10; S8 Table). At reflectance change = 0 (i.e., before dam removal), vegetation cover was similar among depths. With increasing reflectance change, vegetation cover decreased more steeply at deep than at shallow sites.

Plots of vegetation cover versus reflectance change showed more scatter for video data than dive data (Fig 10, top panels). In particular, vegetation cover was high at relatively high reflectance change values (1–2%) for several video data points (Fig 10, top right panel). Most of these points corresponded to measurements made at transects 17 and 18 in August, 2014, where as noted above vegetation cover was considerably higher than at nearby dive sites H1, H2, and K in July 2014 (Fig 5, lower panel).

Linear multiple regression tests revealed that reflectance change and substrate change affected all components of the macroalgal community (Table 4). Reflectance change was a statistically significant predictor of all algae responses and usually explained the highest amount of variation among predictors. Substrate change was significant for all algal response variables except *Agarum* density and explained high percentages of variation for primary cover assemblage and total primary cover. Univariate algal responses were always inversely related to reflectance and substrate change. Initial conditions of depth and percent sand were significant for many response variables, while initial reflectance was only significant for *Agarum* and primary cover. Significant predictors explained relatively high percentages of the variation in algal response (R^2^ range 53–84% for dive data; Table 4).

**Table 4:** Linear multiple regressions for macroalgae.

Response	Predictor; entries are: delta R-squared^*P*-value^ (regression coefficient sign)							R^2^	Adj R^2^
	Initial depth	Initial percent sand	Initial reflectance	Change in reflectance	CR^2^	ID*CR	Substrate change (yes or no)		
**Dive data**									
Brown algae density, assemblage multivariate similarity	8.4***	3.0**	NS	18.5***	3.7***	3.7***	8.9***	56.3	51.9
Total brown algae density^a^	6.1*** (-)	2.8* (-)	NS	14.9*** (-)	5.5*** (+)	3.0** (-)	13.4*** (-)	75.4	72.9
Number of brown algae taxa^a^	4.1** (-)	NS	NS	15.4*** (-)	5.6*** (+)	2.3* (-)	17.8*** (-)	76.6	74.6
Pterygophora density^a^	22.6*** (-)	8.7*** (-)	NS	4.2* (-)	NS	NS	5.1** (-)	60.1	57.5
Saccharina density^a^	3.6* (-)	NS	NS	19.5*** (-)	6.6** (+)	NS	3.9* (-)	65.3	63.1
Agarum density^a^	20.3*** (+)	NS	12.8*** (-)	15.7*** (-)	NS	8.3** (-)	NS	53.7	50.6
Fleshy red algae presence/absence index, assemblage multivariate similarity	5.9***	3.3*	NS	19.7***	4.2**	NS	9.8***	52.8	48.8
Mean fleshy red algae presence/absence index	7.8*** (-)	NS	NS	20.6*** (-)	8.6*** (+)	NS	6.9** (-)	65.8	63.4
Primary cover (%) of small fleshy and encrusting reds and kelp holdfasts, assemblage multivariate similarity	3.5***	15.6***	3.6**	7.8***	NS	NS	15.4***	58.0	54.5
Total primary cover (%)^a^	NS	17.0*** (-)	2.8** (-)	7.5*** (-)	2.5* (+)	NS	20.8*** (-)	76.7	74.7
Secondary cover (%) of algae phyla, assemblage multivariate similarity	6.4***	NS	NS	30.2***	5.1***	3.3***	11.2***	68.1	65.4
Total secondary cover (%)^a^	6.1*** (-)	NS	NS	21.1*** (-)	6.5*** (+)	NS	7.8*** (-)	84.3	83.3
**Video data**									
Vegetation cover (%)	3.2**^b^	NA	Excluded^c^	8.9***^b^	NS	NS	NA	12.0^b^	NA
Kelp cover (%)	NS	NA	Excluded^c^	5.1**^b^	NS	NS	NA	5.1^b^	NA

Non-significant predictors were dropped using backwards selection. Delta R^2^ is the increase in R^2^ obtained (i.e., additional variation explained) when the predictor is added to a model already containing all other predictors. Delta R^2^s do not sum to R^2^. Multivariate tests were conducted for responses designated as “assemblage multivariate similarity”; univariate tests were conducted otherwise. Regression coefficients were not available for multivariate responses. CR^2^ = change in reflectance squared; CR^2^ regression coefficients were always positive indicating a convex curve (apex at the bottom, curve opens up) as shown in Fig 10. ID*CR = the interaction between initial depth and change in reflectance; ID*CR coefficients were always negative indicating that the slope of the inverse relation between algae abundance and reflectance steepened with increasing depth, as shown in Fig 10. The sign of the substrate change coefficient indicates the direction of response variable change for substrate change = “yes” relative to substrate change = “no”. NA = not applicable. *P*-values: NS = not significant.

*** = *P* < 0.001;

** = *P* < 0.01;

* = *P* < 0.05;

^a^Ln(y+1) transformed;

^b^Marginal R^2^s (and delta R^2^ computed from them). They indicate the percentage of variation explained by the fixed effects in the model, but not the random effects, and therefore are not comparable to R^2^s for dive data models which did not include random effects;

^c^Excluded due to collinearity with initial depth (*r* = -0.52).

**Fig 11:**
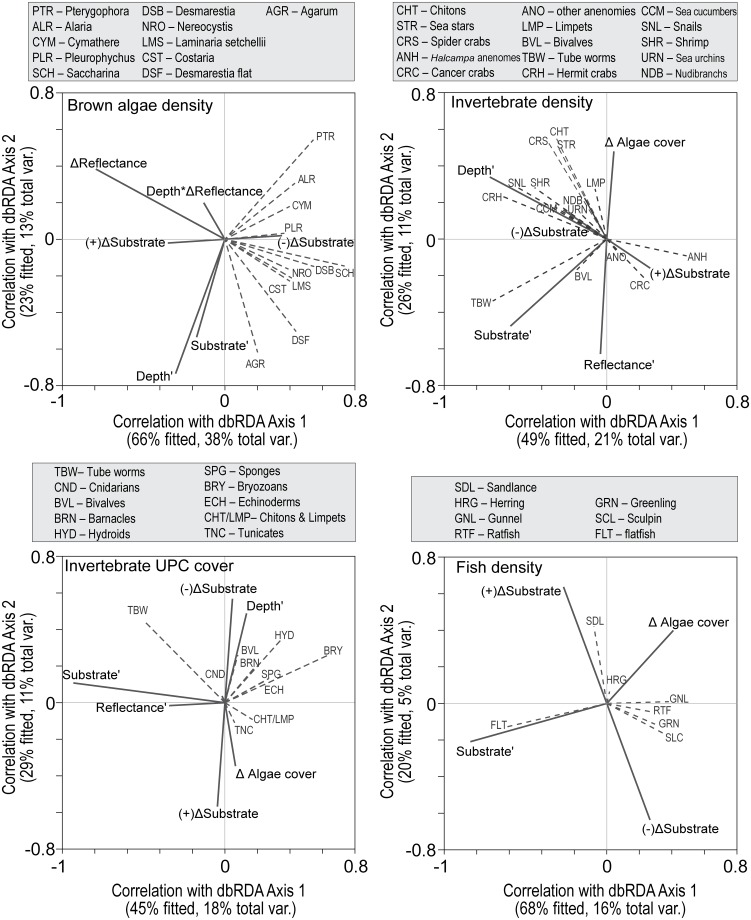
Distance-based redundancy analysis (dbRDA) plots showing relations between assemblages and environment variables. These four plots provide a visualization of the corresponding multivariate multiple regression models in Tables 4 and 5. Predictor vectors (solid lines) indicate the direction and strength of the multiple partial correlation between each environmental variable and each dbRDA axis. The multiple partial correlation for a variable is conditional on the other variables in the model and indicates correlation when the other variables are held constant. Taxon vectors (dashed lines) indicate the direction and strength of the raw (not conditional) correlation between each taxon and each axis. Apostrophes signify initial conditions before dam removal, delta symbols signify change in a variable, and delta +/- signifies categorical change yes (+) or no (-).

Variation in brown algal assemblage was significantly related to all predictors except initial reflectance (Table 4). As shown in a dbRDA ordination of the assemblage model (Fig 11, top left), all individual brown algae taxa were inversely related to reflectance change as evidenced by their vectors pointing away from reflectance change at various angles depending on their relationships to the other predictors. *Agarum* abundance was positively related to depth and was never present at sites where substrate changed and therefore not related to that predictor (Fig 11 top left; Table 4); all other taxa were inversely related to substrate change. *Pterygophora* was inversely related to depth and was less affected by reflectance change than the other taxa (Fig 11, top left; Table 4) probably because its stipes persisted after its blades were damaged. *Saccharina* had a weak inverse relationship with depth and a strong inverse relationship with reflectance change, a response pattern seen in several other taxa (bushy *Desmarestia*, *Nereocystis*, *L*. *setchellii*, *Costaria*) (Fig 11, top left; Table 4).

Total brown algal density, number of brown algal taxa, fleshy red algal assemblage, and secondary cover assemblage showed similar relationships to the predictors as brown algal assemblage (Table 4). Compared to the other algae responses, primary cover assemblage and total primary cover were less strongly related to reflectance change and depth, and more strongly related to substrate change and percent sand (Table 4). The brown algae assemblage did not respond to deposition of sand on gravel differently from deposition of mud on sand (S9 Table). Also, substituting year for reflectance change did not increase model fit for any algae response.

### 4.4 Invertebrate response

#### 4.4.1 Status before dam removal

We surveyed 99 invertebrate taxa for density (Fine analysis group; S6 Table), including at least 147 species (Species present; S6 Table). The 99 taxa were grouped into 24 coarser taxa (Coarse analysis group; S6 Table) for some analyses. The most abundant invertebrates before dam removal were tubeworms, anemones of the genus *Halcampa*, and bivalves (S7 Table). Total invertebrate density averaged 2.7 individuals per m^2^ before dam removal and total primary cover of sessile invertebrates averaged 3.8%, with bryozoans contributing 1.9%, tubeworms 1.4%, and the other categories ≤ 0.2% each (S7 Table).

#### 4.4.2 Spatial and temporal changes

We did not see a significant difference among site-groups in change over time in repeated measures analyses (i.e., the site-group*year interaction was not significant for any invertebrate response, Table 3); this was a major difference from the algal response to dam removal. Further, the factor year was significant for the invertebrate assemblage in repeated measures analyses (true for both the density and primary cover assemblages; Table 3). This finding, in conjunction with the lack of significance for the site-group*year interaction term, suggests a pattern of significant assemblage change over time that was similar at all site-group locations including the control area. Similar change over time at all site-group locations suggests that regional factors rather than dam removal were driving temporal changes in the invertebrate assemblage.

Nevertheless, there was some indication that assemblage change over time differed between the Control site-group and the other site-groups. The trajectory of assemblage change over time appeared to differ between the Control site-group and the other three site-groups (Fig 8), and density assemblage change over time differed significantly between the Control and East site-groups (pair-wise interaction contrasts; Table 3). A difference in change over time between the Control site-group and the other site-groups suggests that dam removal effects contributed to assemblage change. In total, the repeated measures results suggest that invertebrate assemblage change was driven by a combination of regional factors and dam removal effects.

#### 4.4.3 Response to substrate and turbidity changes

Invertebrates responded strongly to substrate change, but in comparison to macroalgae showed weaker and less uniform responses to reflectance change (or the loss of macroalgae cover associated with it). The number of invertebrate taxa decreased when substrate changed, but in contrast to macroalgae, the number of invertebrate taxa did not decrease at Mouth sites without substrate change (Fig 9). Multiple regressions showed that substrate change was significant for all invertebrate response variables, and univariate invertebrate responses were always inversely related to substrate change (Table 5).

**Table 5:** Multiple regressions for invertebrates and fish.

Group	Response	x1, x2	Predictor; entries are: delta R-squared^*P*-value^ (coefficient sign)					R^2^	Adj R^2^	AICc
			x1	x2	Initial depth	Initial percent sand	Substrate change (yes or no)			
Inverte-brates	Density, assemblage multivariate similarity	IR, CR	6.3***	2.0*	9.8***	7.1***	5.2***	35.6	30.3	505.6
		IA, CA	2.1**	2.9**	10.3***	5.4***	5.5***	32.3	26.6	509.0
		**IR, CA**	6.3***	2.8***	9.5***	7.1***	5.6***	**36.5**	**31.2**	**504.7**
	Density, coarser taxonomic grouping, assemblage multivariate similarity	IR, CR	7.5***	NS	11.1***	5.5***	8.7***	38.2	34.2	462.6
		IA, CA	NS	4.2***	10.7***	7.4***	7.8***	34.9	30.6	466.1
		**IR, CA**	7.3***	3.9***	10.4***	6.9***	7.9***	**42.1**	**37.3**	**460.7**
	Total density	IR, CR	14.1*** (+)	NS	15.6*** (+)	4.7* (+)	13.9*** (-)	51.6	48.4	151.8
		IA, CA	NS	8.7** (-)	9.5** (+)	11.9*** (+)	18.7*** (-)	46.2	42.7	158.8
		IR, CA	13.3*** (+)	7.9** (-)	11.0*** (+)	9.9*** (+)	19.9*** (-)	59.5	56.1	142.6
		**IR, Yr**	13.6*** (+)	12.0***	15.8*** (+)	5.8*** (+)	22.2*** (-)	**63.6**	**59.2**	**140.9**
	Number of taxa	**IR, CR**	**NS**	**NS**	13.1*** (+)	**NS**	46.3*** (-)	**58.7**	**57.4**	**136.6**
		**IA, CA**	**NS**	**NS**	13.1*** (+)	**NS**	46.3*** (-)	**58.7**	**57.4**	**136.6**
		**IR, CA**	**NS**	**NS**	13.1*** (+)	**NS**	46.3*** (-)	**58.7**	**57.4**	**136.6**
	Deposition-intolerant invertebrate density^a^	**IR, CR**	4.4* (-)	**NS**	34.2*** (+)	**NS**	24.1*** (-)	**64.6**	**62.9**	**128.7**
		IA, CA	NS	NS	35.4*** (+)	NS	25.6*** (-)	60.2	58.9	134.1
		**IR, CA**	4.4* (-)	**NS**	34.2*** (+)	**NS**	24.1*** (-)	**64.6**	**62.9**	**128.7**
	Tubeworm density	IR, CR	9.7*** (+)	3.1* (+)	15.3*** (+)	21.0*** (+)	7.7*** (-)	63.4	60.3	135.9
		IA, CA	NS	7.0** (-)	11.3*** (+)	26.7*** (+)	8.7*** (-)	60.0	57.3	139.3
		**IR, CA**	6.8*** (+)	6.4** (-)	12.5*** (+)	24.4*** (+)	9.3*** (-)	**66.7**	**64.0**	**129.6**
	Halcampa density	**IR, CR**	13.2*** (+)	**NS**	19.9*** (-)	6.3** (-)	5.5** (-)	**57.2**	**54.4**	**143.7**
		IA, CA	NS	NS	22.1*** (-)	5.0* (-)	4.7* (-)	44.0	41.3	159.0
		**IR, CA**	13.2*** (+)	**NS**	19.9*** (-)	6.3** (-)	5.5** (-)	**57.2**	**54.4**	**143.7**
	Bivalve density	IR, CR	NS	NS	NS	NS	NS	----	----	----
		IA, CA	NS	9.0* (-)	NS	NS	7.2* (-)	12.9	10.1	185.8
		IR, CA	NS	9.0* (-)	NS	NS	7.2* (-)	12.9	10.1	185.8
		**IR, Yr**	**NS**	21.8**	**NS**	**NS**	11.7** (-)	**25.7**	**20.8**	**180.1**
	Cancer crab density	IR, CR	8.7* (+)	NS	11.7** (-)	NS	NS	21.2	18.7	179.2
		IA, CA	NS	NS	12.6** (-)	NS	NS	12.6	11.2	183.8
		IR, CA	8.7* (+)	NS	11.7** (-)	NS	NS	21.2	18.7	179.2
		**IR, Yr**	9.1** (+)	11.2*	10.9** (-)	**NS**	7.0* (-)	**34.8**	**28.1**	**176.6**
	Pycnopodia density	IR, CR	NS	NS	NS	NS	10.8** (-)	10.8	9.4	185.1
		IA, CA	NS	NS	NS	NS	10.8** (-)	10.8	9.4	185.1
		IR, CA	NS	NS	NS	NS	10.8** (-)	10.8	9.4	185.1
		**IR, Yr**	**NS**	19.8*	**NS**	**NS**	5.1* (-)	**30.5**	**26.0**	**175.7**
	Primary cover (%) of sessile and encrusting species, assemblage multivariate similarity	IR, CR	3.7**	8.1***	3.8**	11.9***	8.1***	37.9	32.7	471.4
		IA, CA	2.5*	10.4***	5.1***	10.3***	7.3***	39.3	34.2	469.9
		**IR, CA**	2.7*	9.8***	4.8***	13.1***	7.3***	**39.6**	**34.5**	**469.6**
	Total primary cover (%)	**IR, CR**	**NS**	20.0*** (+)	8.7** (+)	**NS**	30.8*** (-)	**49.9**	**47.4**	**151.7**
		IA, CA	NS	10.2** (-)	8.5** (+)	NS	27.2*** (-)	40.0	37.1	163.6
		IR, CA	NS	10.2** (-)	8.5** (+)	NS	27.2*** (-)	40.0	37.1	163.6
Fish	Density, assemblage multivariate similarity	IR, CR	NS	3.0*	NS	9.5***	4.7**	19.9	16.1	484.5
		**IA, CA**	**NS**	5.1***	**NS**	11.4***	4.8**	**22.0**	**18.2**	**482.7**
		**IR, CA**	**NS**	5.1***	**NS**	11.4***	4.8**	**22.0**	**18.2**	**482.7**
	Density, coarser taxonomic grouping, assemblage multivariate similarity	IR, CR	NS	NS	NS	9.2***	7.6***	18.5	16.0	472.0
		**IA, CA**	**NS**	5.8***	**NS**	11.3***	6.2**	**24.3**	**20.7**	**469.4**
		**IR, CA**	**NS**	5.8***	**NS**	11.3***	6.2**	**24.3**	**20.7**	**469.4**
	Total density	IR, CR	NS	NS	NS	NS	NS	----	----	----
		**IA, CA**	6.2* (-)	**NS**	**NS**	**NS**	**NS**	**6.2**	**4.7**	**188.4**
		IR, CA	NS	NS	NS	NS	NS	----	----	----
	Number of taxa	**IR, CR**	**NS**	**NS**	**NS**	**NS**	9.9* (-)	**9.9**	**8.5**	**185.8**
		**IA, CA**	**NS**	**NS**	**NS**	**NS**	9.9* (-)	**9.9**	**8.5**	**185.8**
		**IR, CA**	**NS**	**NS**	**NS**	**NS**	9.9* (-)	**9.9**	**8.5**	**185.8**
	Sculpin density	IR, CR	NS	NS	NS	7.9* (-)	5.9* (-)	16.5	13.9	183.0
		IA, CA	NS	NS	NS	7.9* (-)	5.9* (-)	16.5	13.9	183.0
		IR, CA	NS	NS	NS	7.9* (-)	5.9* (-)	16.5	13.9	183.0
		**IR, Yr**	**NS**	13.9*	**NS**	10.5** (-)	**NS**	**24.5**	**19.6**	**181.1**
	Flatfish density	IR, CR	NS	6.8* (+)	NS	30.8*** (+)	NS	33.3	31.2	168.2
		**IA, CA**	**NS**	10.7** (-)	**NS**	35.1*** (+)	**NS**	**37.2**	**35.2**	**164.3**
		**IR, CA**	**NS**	10.7** (-)	**NS**	35.1*** (+)	**NS**	**37.2**	**35.2**	**164.3**
	Sand lance density	**IR, CR**	**NS**	**NS**	**NS**	**NS**	15.4** (+)	**15.4**	**14.1**	**181.6**
		**IA, CA**	**NS**	**NS**	**NS**	**NS**	15.4** (+)	**15.4**	**14.1**	**181.6**
		**IR, CA**	**NS**	**NS**	**NS**	**NS**	15.4** (+)	**15.4**	**14.1**	**181.6**

We tested whether response variables were more strongly associated with reflectance or algal cover. IR = initial reflectance, CR = change in reflectance, IA = initial algal cover, CA = change in algal cover. Three combinations of initial conditions (x1) and change in conditions (x2) are shown. The fourth (x1 = IA, x2 = CR) never fit better than at least one of the other three combinations. AIC_c_ indicates which model fit best for a particular response (lower AIC_c_ means better fit) but cannot be compared among responses. A model with x2 = year fit better than the best fitting model with x2 = CR or CA for some responses; these models are included in the table (Yr = year). Better fit for models with year may indicate spatially broad effects unrelated to dam removal, for example recruitment or disease (see text). The best fitting model for each response is bolded. Regression coefficient sign is not shown for year because for each model, year had three regression coefficients associated with it. All univariate responses were ln(y+1) transformed. All else is the same as in Table 4.

^a^Invertebrates that responded negatively to substrate change but did not respond to change in reflectance or algae cover (Fig 11).

Reflectance change and algal cover change were significant predictors for some invertebrate response variables (Table 5). When significant, algal cover change was usually a stronger predictor than reflectance change (i.e., greater delta R-squared and better fit for the model that included it; Table 5). In multivariate tests, the density and primary cover assemblages were significantly related to algal cover change. In univariate tests, significant inverse relationships with algal cover change (and positive relationships with reflectance change) were found for tubeworm density and total primary cover. Initial conditions of depth, percent sand, and reflectance consistently explained substantial percentages of invertebrate response variation. Best-fitting multiple regression models for invertebrates generally explained lower percentages of total variation (R^2^ range 26–67%; Table 5) than did best fitting models for macroalgae.

Multivariate multiple regression results were similar for the fine and coarse taxonomic assemblages (S6 Table); best fitting models for both included change in algal cover and substrate, and initial reflectance, depth, and percent sand as predictors (Table 5). Several of the coarse taxa were inversely related to substrate change and positively related to depth (Fig 11, top right, taxon vectors in the top-left quadrant). We assigned these taxa to a “deposition-intolerant” group and summed their densities (excluding sea stars for reasons given below). Total density of deposition-intolerant taxa was strongly related to depth (positively) and substrate change (negatively), and weakly related to initial reflectance (negatively) (Table 5). Tubeworms showed a different response pattern. In addition to being negatively related to algal cover change, tubeworms were positively related to initial percent sand, positively related to initial reflectance, and weakly related to substrate change (negatively) and depth (positively) (Fig 11, top right; Table 5). Halcampa anemonies showed a third response pattern primarily driven by a strong inverse relationship with depth (Fig 11, top right; Table 5). The number of invertebrate taxa was positively related to depth and very strongly negatively related to substrate change (Table 5), the latter probably attributable to the loss of many deposition-intolerant taxa.

Best fitting models for the primary cover assemblage and density assemblage included the same predictors; however, algal cover change and initial percent sand were stronger predictors for the primary cover assemblage than for the density assemblage (Table 5). Primary cover of tubeworms, bryozoans, and hydroids was negatively related to both algal cover change and substrate change (Fig 11, bottom left). The relationship between primary cover and initial percent sand was positive for tubeworms but negative for hydroids and bryozoans.

**Fig 12:**
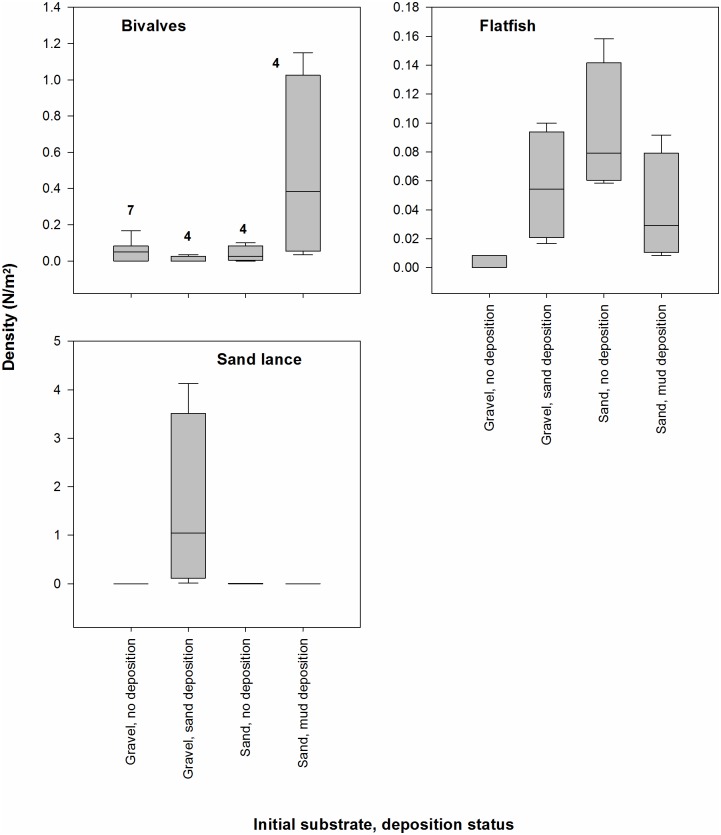
Density of taxa that responded differently to two types of substrate change: Deposition of sand on gravel-cobble and deposition of mud on sand. Only the five sites where substrate changed (Fig 3) are included. Numbers next to boxes in top-left panel indicate sample size (number of site-years).

The invertebrate density assemblage showed a marginally different response between deposition of sand on gravel and deposition of mud on sand (*P* = 0.06 for the interaction between initial substrate and substrate change; S9 Table). Bivalves were the only single invertebrate taxon to respond differently to the two deposition scenarios. Bivalves decreased when sand deposited on gravel but increased when mud deposited on sand (Fig 12).

#### 4.4.4 Response to regional drivers

Bivalves and cancer crabs were better fit by models that included year instead of algal cover change or reflectance change (Table 5), which may indicate interannual variability in spatially broad effects unrelated to dam removal. With the effect of year isolated from effects of other significant predictors, bivalve density was highest in 2013 (S4 Fig), driven mainly by a spike across the study area in 2013 in the density of the most abundant bivalve, *Mya truncata*, a pattern suggesting widespread recruitment of this species. Cancer crab density was higher in 2013 and 2014 than previously (S4 Fig). Density spiked at several sites for juvenile *Metacarcinus magister* in 2013 and for *Cancer gracilis* and *C*. *oregonensis* in 2014. Total density of invertebrates was also better fit by a model that included year instead of reflectance or algal cover change. Total invertebrate density increased from before dam removal to 2013, then leveled off in 2014 (S4 Fig). Recruitment of bivalves and cancer crabs likely contributed to this pattern.

We first observed symptoms of sea star wasting disease (SSWD) [87] in 2014. *Pycnopodia helianthoides*, a species particularly susceptible to SSWD [87], was the most abundant sea star in our study area before 2014. We therefore tested *P*. *helianthoides* for effects of year versus reflectance or algal cover change and found a better fit for year (Table 5). *P*. *helianthoides* density decreased substantially in 2014 (S4 Fig) consistent with the pattern of SSWD mortality in the broader Salish Sea region [88].

### 4.5 Fish response

#### 4.5.1 Status before dam removal

We surveyed the density of 28 fish taxa that included 53 species (S6 Table). We grouped these taxa into 16 coarser taxonomic groups for some analyses (S6 Table). Before dam removal the most abundant fish were herring, sand lance, sculpin, ratfish, gunnels, flatfish, and greenling, and total fish density averaged 0.4 individuals per m^2^ (S7 Table).

#### 4.5.2 Spatial and temporal changes and response to regional drivers

Change over time did not differ among site-groups for any fish response in repeated measures analyses (Table 3). The main effect of year was significant for the fish assemblage (Table 3), suggesting that regional factors were driving change rather than dam removal (Fig 8). The best fitting multiple regression model for sculpin included year rather than reflectance change or algae cover change (Table 5). Interannual variation in sculpin density showed a pattern of alternating increases and decreases (S4 Fig) that was primarily driven by variation in the density of buffalo sculpin, the most abundant sculpin observed in our surveys.

#### 4.5.3 Response to substrate and turbidity changes

The relationship between fish and substrate change was less consistent than it was for invertebrates. Similar to invertebrates, the fish assemblage changed with substrate change and the number of fish taxa present was negatively related to substrate change (Table 5). However, flatfish density was unrelated to substrate change and sand lance density was positively related. Algal cover change better explained fish response than reflectance change, but it was only significant for the multivariate assemblage and flatfish density (Table 5). Initial percent sand was often a significant predictor of fish response, but depth and initial reflectance never were (Table 5). Best fitting models generally explained lower percentages of variation for fish responses (R^2^ range 6–37%) than for invertebrates or algae.

Best fitting multivariate multiple regression models for both the fine and coarse assemblages (S6 Table) included change in algal cover and substrate, and initial percent sand (Table 5). Flatfish density was related to algal cover change (negatively) and percent sand (positively), whereas only substrate change was significant for sand lance (Fig 11, bottom right; Table 5). Greenling, gunnels, ratfish, and sculpin were negatively related to percent sand (Fig 11, bottom right; Table 5).

Flatfish and sand lance responded to deposition of sand on gravel differently than deposition of mud on sand (S9 Table; Fig 12). Flatfish increased when sand deposited on gravel-cobble but decreased when mud deposited on sand (Fig 12). In contrast, sand lance increased only when sand deposited on gravel; they were nearly absent otherwise (Fig 12). Other fish did not respond differently to the two substrate change types (S9 Table).

## 5. Discussion

The degree to which sediment deposition drives changes in benthic marine habitats and biological communities is an important scientific and management issue, particularly in regards to anthropogenic sources like those derived from a large-scale dam removal [1, 4, 89]. During the simultaneous removal of two dams on the Elwha River that was phased over three years, decades worth of annual sediment load was released that changed nearshore subtidal communities. Sediment entering the coastal system increased suspended sediment concentrations and formed persistent deposits on the seafloor in some areas. Community change differed depending on whether elevated suspended sediment was accompanied by sediment deposition or not. Spatial and temporal patterns of suspended and deposited sediments determined where and when each type of community change occurred.

Although suspended sediment was most elevated near the river mouth, it was higher than before dam removal at least 5 km west and 9 km east of the river mouth and offshore beyond our deepest sites. Persistent sediment deposits were confined to a smaller area, accumulating on the seafloor roughly 2 km to either side of the river mouth and 0.5 km offshore. Sand deposited on gravel substrate off the mouth and to the east, and mud deposited on originally sandy substrate to the west. Suspended sediment increased in the first year of dam removal and remained high during the study period, but deposition mostly occurred in the second and third years of dam removal.

**Fig 13:**
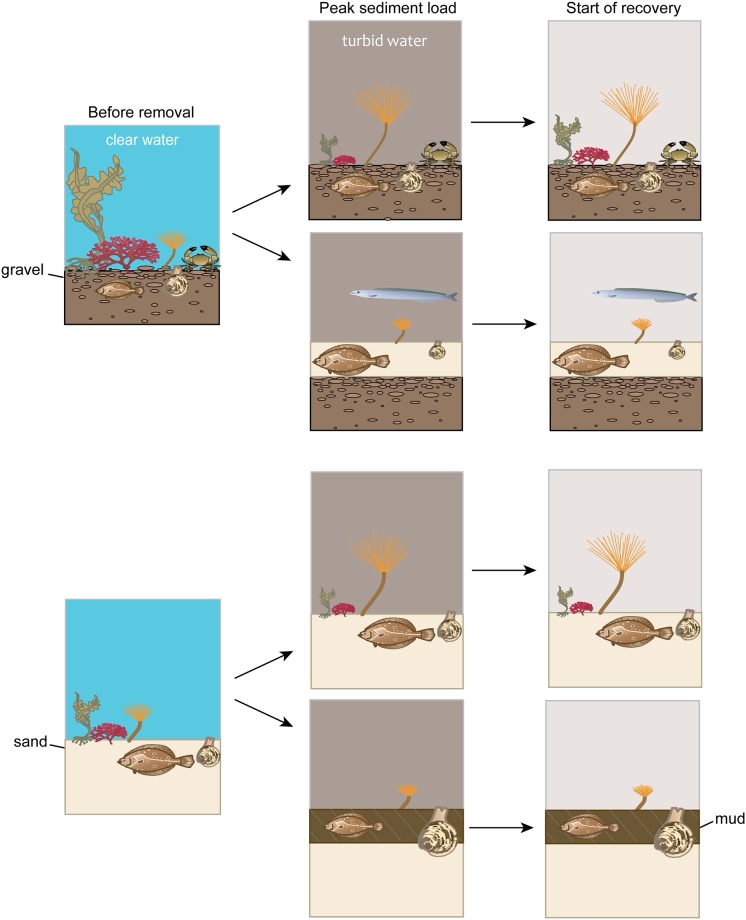
Conceptual model of nearshore subtidal community responses to sediment deposition and elevated turbidity associated with dam removal on the Elwha River. The size of algae, invertebrate, and fish symbols is proportional to their abundance. Plant and animal images were courtesy of the Integration and Application Network, University of Maryland Center for Environmental Science (ian.umces.edu/imagelibrary/). Image creators were: Crab: Kim Kraeer and Lucy Van Essen-Fishman; flatfish: Jane Thomas; algae, sand lance, and clam: Tracey Saxby.

Biological responses primarily depended on the initial substrate, whether turbidity increases were accompanied by sediment deposition, and what type of material (i.e., sand or mud) was deposited (Fig 13). The main biological response to increased turbidity was a large reduction in algae, especially on gravel substrate where they were abundant before dam removal (Fig 13). Most invertebrates and fish were unaffected by increased turbidity; however, tubeworms, hydroids, bryozoans, and flatfish showed increases that could have been due to decreased algal cover rather than increased turbidity (i.e., an indirect effect of turbidity rather than a direct effect; see Discussion in Section 5.1 below). Deposition of sand on gravel substrate resulted in wholesale community change (Fig 13): any algae that persisted under increased turbidity conditions in year 1 were eliminated when sediment deposition occurred; hard substrate-associated invertebrates and fish were eliminated or greatly reduced; bivalves and tubeworms decreased; and flatfish and sand lance increased. Community changes were less extreme where mud deposited on sand (Fig 13). Algae were eliminated but their abundance had been low before dam removal, tubeworms and flatfish decreased moderately, and bivalves increased.

In the third year of dam removal algae partially recovered on gravel substrate in some areas outside the sediment deposition zone (Fig 13). We hypothesize that delayed algal recruitment associated with decreased turbidity and increased seafloor light was responsible (see Discussion in Section 5.1 below). If so, it would mark the beginning of recovery from turbidity impacts as shown in the top-right panel of Fig 13. We did not detect an invertebrate or fish response to the turbidity reduction.

Our results are discussed in more detail below. Limitations of some of our methods including the BACI study design, direct observation by divers, and using light reflectance as a proxy for suspended sediment concentration are discussed in S2 Text.

### 5.1 Response to turbidity

It is likely that light attenuation associated with increased suspended sediment accounted for the inverse relationship between algae and reflectance (i.e., our proxy for suspended sediment and turbidity), and the large decrease in algae outside of the sediment deposition zone. Many studies have documented the negative effect of reduced light on kelp survival, particularly the gametophyte and juvenile sporophyte stages [90–92]. Although some kelp species can survive in low light conditions [93, 94], reproduction and growth are reduced [95]. Given favorable temperatures and adequate nutrients, the amount of light reaching the seafloor sets the maximum depth limit of kelp and other benthic algae [96, 97].

We observed partial recovery of algae on the east side of the Elwha River delta in 2014, the third year of dam removal. Relatively high percent cover of algae was observed on 29 August 2014 at towed video transects 17 and 18 (Fig 5). Three nearby dive sites (H1, H2, and K) had much lower algal cover (Fig 5) during surveys made more than a month earlier (20–23 July 2014). We resurveyed dive site H1 on 19 August 2014, and over that nearly one month period, cover of algae increased from 17% to 63%, brown algal density increased from 2 to 3.4 plants per m^2^, and number of brown algal taxa present increased from 3 to 7, demonstrating recruitment and growth of algae between late July and late August at site H1. This late season recruitment likely accounts for the discrepancy in algal abundance between dive data and towed video transect data. Sediment flux in the river, likely indicative of sediment entering marine waters, decreased to low levels (< 500 tonnes per day) by June 2014, at least three weeks earlier than in 2012 or 2013. Perhaps suspended sediment was reduced early enough in 2014 to allow sufficient light for recruitment of newly settled algal propagules or development of microscopic stages that were already present. Delayed development of microscopic stages of kelp and other macroalgae can occur during periods of unfavorable conditions [98]. This highlights the capacity of algae to respond quickly to changes in turbidity and light availability and suggests that algae could recover as sediment loads to marine waters decrease.

The non-linear relationship between algal cover and reflectance change, showing steep declines in algal cover as reflectance increased to about 1% above pre-dam removal levels but less change in algal cover with further reflectance increases (Fig 10), suggests that the minimum amount of light needed for algae wellbeing was reached at the inflection point in the curve (about a 1% reflectance increase). We did not measure the amount of light reaching the seafloor and do not know the light level that corresponded to a 1% reflectance increase, but work is underway to better understand relationships between suspended sediment concentrations and light attenuation, and between light and algal survival, growth, and recruitment in the Elwha system. The depth-dependency in the relationship between algae cover and reflectance change has several implications. The similarity in algal cover among depths at zero reflectance change (Fig 10) suggests that before dam removal, light did not limit algae even at our deep sites because if light had been limiting, algal cover would have been less at deep than at shallow sites due to light attenuation with depth. With increases in reflectance, the steeper decline in algal cover at deep than at shallow sites (Fig 10) suggests that light became limiting to algae sooner (i.e., at smaller reflectance change increases) at the deep sites. This would be expected because for a given increase in surface turbidity, less light would reach the seafloor at the deep sites due to light attenuation with depth. A lingering question concerns how algae were able to persist at low levels at shallow sites even at relatively high reflectance increases (up to 3%; Fig 10). Apparently some light still reached the bottom at the shallow sites, perhaps because of less depth-related light attenuation, shallowing at low tide, or short term clearing due to current-driven plume dynamics.

It is possible that algal recruitment and survival were hindered by benthic scouring and ephemeral deposition [99, 100] in addition to reduced light. Tolerance to sediment deposition is dependent on algal morphology. Species that propagate vegetatively or regenerate from basal thallus parts are the most resistant to sedimentation effects [101–103]. Most of the algal species in our study area are prostrate, bladed species that are more susceptible to scouring and burial [102, 104]. In addition, while kelp gametes can survive for long periods in some settings [90], most only live a few weeks [92, 105], decreasing their chances of survival when conditions are unfavorable (e.g., subject to ephemeral sedimentation) for long periods of time. Benthic photos taken every four hours from a tripod near dive site E1 during parts of 2012 and 2013 showed that a high percentage of photos were characterized by ephemeral sediment deposition on the seafloor and (or) turbid conditions [78]. In most instances ephemeral deposition lasted less than four hours, highlighting the high frequency of sediment deposition and erosion in this system. Scouring likely occurred as deposited sediment was resuspended by high velocity tidal currents, particularly east of the river mouth [43].

Tubeworm and flatfish density, and total primary cover of sessile invertebrates, increased in response to suspended sediment increases and/or algal cover losses. The inverse relationship between sessile invertebrates and algae may have resulted because prostrate kelp and other understory algae that can cover and abrade them were removed [106]. The positive relationship between sessile invertebrates and suspended sediment may have been due to an enhanced food supply. Organic material released from the reservoirs when the dams were removed could have increased the amount of food available, even though it may have been of a lower quality than marine-derived organic matter [107, 108]. Being sessile, tubeworms can increase their abundance in an area through reproduction but not immigration. One of the most common tubeworms at our sites, *Eudistylia vancouveri*, can recruit and build full sized (i.e., visible to divers) tubes within one year (Steven Fradkin, NPS, personal communication), suggesting that the observed increase in tubeworm abundance is biologically feasible given the short time frame of our study. Mobile organisms, like flatfish, likely immigrated and recruited to the new habitat, as we saw a wide size-range of individuals ranging from small juveniles to full grown adults. Flatfish may prefer bare substrate over heavy algal cover, although juveniles are sometimes associated with moderate densities of tubeworms and other habitat structure [109].

Most invertebrates and fish (other than tubeworms, other sessile invertebrates, and flatfish) were unaffected by increased suspended sediment or algal cover loss. We expected species dependent on benthic algae for food [110–112] or other functions [113–115] would decrease in response to algal cover loss, but that did not occur. The effects of algal cover loss on invertebrates and fish may have been sub-lethal, so our study may not have been long enough to capture population-level changes. Also, most kelp and other fleshy algae die back in winter and therefore were not present in that season even before dam removal, so it is possible that animals were capable of using other sources of food and cover in the absence of algae.

### 5.2 Response to sediment deposition

Persistent accumulations of deposited sediments fundamentally altered benthic habitat by burying and replacing the original substrate. Benthic algae were mostly eliminated because the rocky substrate they need for survival and recruitment was lost [116, 117]. Invertebrates and fish responded less uniformly to sediment deposition. Species that required (e.g., for attachment) or strongly preferred hard substrate were eliminated or greatly reduced in abundance, driving down taxa richness. A smaller group of species showed some resilience to sediment deposition, maintaining populations but at lower abundances than before dam removal, whereas few species increased in abundance. The overall decrease in diversity associated with deposition supports observations by other investigators [16, 24–27].

For some species, the response to sediment deposition depended on whether sand deposited on gravel/cobble or mud deposited on sand. Observations of sand lance by divers were infrequent near the Elwha River mouth prior to dam removal, but afterwards were common in or near newly deposited sand (Fig 12). Median grain size of the new sand east of the river mouth averaged 0.45 mm (sites D1, E1 and F1; S4 Table), nearly within the grain size range (0.5–1.0 mm) preferred by sand lance for burrowing [118]. Interestingly, though, sand lance were not observed on the pre-dam removal sand deposit west of the mouth (Fig 12) even though grain size was similar (mean of median at sites C1 and C2 = 0.46 mm in 2012, before deposition of mud occurred; S4 Table). Perhaps the more compacted sand did not facilitate burrowing and held less oxygenated interstitial water [118]. Flatfish abundance increased where sand deposited on gravel but decreased where mud deposited on sand (Fig 12), suggesting a preference for sand over gravel or mud. Bivalve abundance decreased where sand deposited on gravel, perhaps because bivalves were trapped in the compact gravel and unable to move upward into the new sand [119, 120]. Contrary to previous studies [12, 121], bivalves increased where mud was deposited on sand (Fig 12), perhaps from a combination of vertical movement (from the old sand into the new mud) and recruitment.

Unfortunately, we did not have sufficient resources to study responses of infauna other than species visible to divers at the substrate surface. Effects of sediment deposition on pre-existing infauna and colonization of the new sediment deposits by infauna would have been particularly interesting. These questions could guide the development of hypotheses for future studies.

### 5.3 Response to regional drivers

Although increased turbidity and sediment deposition associated with dam removal clearly accounted for dramatic vegetation losses near the river mouth, our results also suggest that lower magnitude decreases in algal abundance occurred outside of the immediate vicinity of the Elwha River. Algal density, primary cover and secondary cover decreased at dive sites in the Control site-group near Green Point (Figs 5 and 7), which did not experience deposition or elevated suspended sediment (Fig 4). Control areas for the towed video surveys, at Dungeness Bluffs and Crescent Bay (Fig 1), also showed low magnitude decreases in algal cover (Figs 5 and 6), predominantly in 2013 and 2014. Long-term monitoring of canopy-forming kelp suggests a similar pattern of severe declines in the Elwha vicinity and relatively lower magnitude declines in other areas on the Washington Coast and Strait of Juan de Fuca (Helen Berry, WA DNR, unpublished data).

Variation in the abundance of several invertebrates and fish was better explained by models that included year as a predictor instead of reflectance change or algae cover change. This suggests that interannual variability in recruitment, disease, or other factors, rather than dam removal, may have been at least partially responsible for interannual abundance changes in our study area. Life cycles of most invertebrates and fish include a pelagic larval stage that facilitates widespread dispersal, and often results in high interannual variability in recruitment due to effects of environmental variability on larval survival [122]. Spikes in the abundance of some bivalve, cancer crab, and sculpin species in particular years and across the study area suggested that recruitment contributed to the interannual variability of those taxa. Sea star wasting disease appeared in Strait of Juan de Fuca populations in late 2013 after our summer surveys. Abundances of several sea stars, especially *P*. *helianthodes*, were greatly reduced in our 2014 surveys (S4 Fig, middle right). *P*. *helianthodes* and other sea stars affected by SSWD are important predators of many invertebrates, so any invertebrate increases in 2014 could have been due to a lack of predatory sea stars. We observed a slight increase in total invertebrate density in 2014; however the increase in 2014 was much smaller than the increase in total invertebrate density in 2013 (S4 Fig, top left) which could not be attributed to SSWD.

Bottom water temperature, wave energy flux, and freshwater flux in the study area were relatively similar before and during dam removal (S2 Fig) and therefore probably not responsible for most of the observed community changes. Two anomalously high temperature spikes in early fall and winter of 2013 (S2 Fig) influenced the entire region [123] and could have contributed to the region-wide interannual variation in recruitment and disease described above.

### 5.4 Effect of initial conditions

Initial substrate (characterized in our analyses by initial percent sand) and initial depth accounted for large proportions of the variation in many algal, invertebrate, and fish response metrics. During our study, depth only changed appreciably at one site (D1) and lasting substrate change only occurred at five sites (Fig 3). Even so, initial substrate and depth remained important structuring agents of the benthic community throughout the period of increased sediment load as might be expected given that substrate and depth are known to strongly influence benthic communities [60, 124].

More surprisingly, initial reflectance (i.e., turbidity before dam removal) was an important predictor for several invertebrate responses. Notably, densities of tubeworms, halcampa anemonies, and cancer crabs were positively related to initial reflectance (Table 5). Initial reflectance was not correlated with initial percent sand (*r* = 0.07), indicating that areas with high initial reflectance did not correspond to depositional areas. We hypothesize that tubeworms and halcampa anemonies may have benefited from turbidity-enhanced food supply. Unlike depth and substrate, reflectance changed (increased) at most sites during dam removal, yet the effect of initial reflectance on invertebrates persisted. Initial reflectance showed a moderate inverse correlation with reflectance change (*r* = -0.30) that was primarily due to areas with high initial reflectance showing relatively little reflectance change during dam removal. These sites may have experienced high turbidity levels before and during dam removal and remained good habitat for some of the invertebrates.

### 5.5 Implications for dam removal

Although dam removal has become increasingly common [35], less than 10% of dam removal projects have been scientifically evaluated and few of these are long-term studies [125]. To our knowledge, this is the first dam removal study to evaluate coupled bio-physical changes in the marine environment, as most others have been restricted to estuaries [38, 45, 48, 126, 127] or have looked exclusively at physical changes [45, 48]. The Elwha dam removal project is unique among dam removals, due to the size of the dams and the massive amount of sediments released into the coastal environment, creating physical disturbance to the ecosystem on par with large-scale natural disturbance events like typhoons, volcanic eruptions, and wildfires [89]. The link between dam removal and marine ecosystem response seen in the Elwha nearshore environment could also occur in other coastal dam removal projects, but the duration and level of changes will depend on stored reservoir sediment and its composition, duration of impact (i.e., time period of dam removal), timing of sediment release, and conditions of the receiving ecosystem. For example, projects that release mostly fine sediment over a short period of time and without significant substrate changes to marine environment would be expected to have less impact than projects that significantly alter substrate composition, occur over longer time periods, or co-occur with seasonal peaks in growth or recruitment. Because of the relatively short time frame of our monitoring, it is likely that further changes to the Elwha nearshore environment, including recovery of some algal populations, is possible as the ecosystem recovers from sediment disturbance in the short-term and adjusts to reconnected sediment supply in the long-term. The recovery of these areas, including whether the dam removal effects are short term or long lasting, will be the focus of future research efforts.

### 5.6 Community resiliency

For nearly a century, over 90% of the natural sediment supply of the river was sequestered behind the dams [64]. A reason for removing the dams was to replace this chronic sediment deficit with the natural sediment budget supplied by the river. Yet, to achieve this restoration it was necessary to create a large-scale pulse disturbance, with a massive release of sediment [48]. Despite this large sediment influx, nearshore subtidal communities proved resilient. Invertebrates and fish did not show a significant negative effect of dam removal except where sediment accumulated near the river mouth, and even there soft sediment-adapted species quickly established. Although algae did show a significant negative impact, it is likely a short-lived one and recovery appears to be well underway. This resilience of the marine ecosystem is similar to the resilience seen in freshwater systems following dam removal [35], where negative dam removal effects have been largely short term.

A mechanism that may have facilitated community resilience was the hydrodynamics of the system. Strong tidally-driven currents reoccur regularly over daily and fortnightly (spring versus neap) tidal cycles [43, 45, 50]. These currents restricted persistent deposition to areas near the river mouth or where current speeds were lower (i.e., Freshwater Bay) [43, 45]. Outside of these areas, any deposited sediments were quickly resuspended and transported away, with the long term consequence of maintaining the substrate near its original state [43, 45, 78]. In the short term, organisms had to deal with periodic burial and scour [43, 78], but this did not measurably impact the invertebrates or fish. Much of the very fine sediment entering the system was immediately transported away in the buoyant river plume [45, 48]. The plume was spatially extensive but dissipated when fine sediment entering the system decreased. Even where sediment deposits formed, the flushing effect of currents may have minimized anoxia [23], thereby facilitating habitability.

Algae and animals employed completely different strategies to cope with the sediment influx. Algae were very sensitive to aspects of the plume, almost certainly light reduction but probably also ephemeral burial and scour where it occurred, and declined over large areas, but were capable of rebounding very quickly with clearing. In contrast, animals maintained themselves throughout the sediment influx and in some cases even benefited. A key for all of the algal species and many of the animals was maintenance of the original substrate over large areas. The net result was a high level of whole-community resilience.

We provide this discussion of how local hydrodynamic conditions influenced community resiliency in part to give context for comparison with other systems. Communities in systems ranging from sheltered estuaries [12] to high energy open coasts dominated by waves rather than tidal currents [27, 128] exhibited different levels of resiliency to sediment influxes, in part because of different hydrodynamic regimes.

## 6. Conclusions

The large amount (> 10 Mt) of sediment released into marine waters during removal of the Elwha River dams altered the benthic community. The characteristics of community response differed depending on the context of sedimentation. The two main types of sediment effects were increased turbidity and sediment deposition. Turbidity increases had a greater spatial footprint than formation of sediment deposits, allowing us to separate how these two physical changes affected the community. This might have relevance to future dam removal projects occurring in coastal areas, but might also be more broadly applicable to any scenario of increased coastal sedimentation effects on marine benthic communities.

The greatest community change in response to elevated suspended sediment alone was a large decrease in macroalgae, predominantly kelp and foliose red algae. Macroalgal abundance was inversely related to suspended sediment increases in a manner that suggested a suspended sediment limit to survival. Light reduction from elevated suspended sediment likely caused macroalgal declines [90], but ephemeral deposition and scour probably contributed [43, 78]. Macroalgae also showed a capacity to rebound quickly in response to decreased suspended sediment, as exemplified by their partial recovery through seasonally late recruitment and growth near the completion of dam removal.

Some sessile invertebrates, particularly tubeworms, and flatfish increased in response to increased suspended sediment or the macroalgal loss associated with it. This could have been due to relaxed competition with macroalgae for space [106] or enhanced food supply, perhaps from particulate organic matter released from the former reservoirs. Other invertebrates and fish were unaffected by increased turbidity or algal loss. No invertebrates or fish declined detectably, contrary to other studies showing animal declines associated with reduced macroalgae [113, 114].

Persistent sediment deposition greatly altered the benthic environment through burial and conversion of substrate to finer-grained material. Macroalgae were nearly eliminated, presumably because the hard substrate they need for attachment was lost [1]. All invertebrate taxa declined, as did invertebrate taxa richness in keeping with other studies [16, 24, 26, 27]. Bivalves were the most resilient invertebrate group, increasing in response to a particular type of deposition (see below). Most fish also declined, as did fish species richness, but flatfish were indifferent to deposition and sand lance increased.

Community response to deposition depended to some extent on the depositional scenario. Where sand deposited on gravel, formerly abundant macroalgae were eliminated, and hard substrate-associated invertebrates and fish including chitons, limpets, spider crabs, greenlings, gunnels, and sculpins were also eliminated or greatly reduced; however, the sand-adapted flatfish and sand lance increased. Tubeworms, bivalves, and flatfish were abundant on initially sandy substrate. Where mud deposited on sand, tubeworms and flatfish declined but remained present, and bivalves increased in contrast to other studies [12, 121].

Strong tidally-driven currents limited persistent sediment deposition to areas near the river mouth and maintained the original gravel-cobble substrate over large areas [43, 45]. Algae declined in these areas due to increased suspended sediment but were capable of rebounding quickly when the water cleared. Invertebrates and fish did not decline in these areas. The net result was a relatively high level of whole-community resiliency. Even where sediment deposits formed, some soft sediment-adapted invertebrates and fish quickly immigrated or recruited.

## Supporting information

S1 Fig Sampling effort for dive sites (Fig 1) by year and site, as well as taxa richness for algae and invertebrates. (TIF)

S2 Fig Environmental conditions. (A) Elwha River mean daily discharge and (B) sediment flux (cumulative and daily average, in tonnes) estimated at USGS River gage 12046260 (rkm 5), (C) mean daily wave energy flux at NOAA Hein Bank buoy (Fig 1), and (D) mean daily water temperature at a depth of 6m MLLW at site E1 near the Elwha River mouth.
(TIF)

S3 Fig Mean density of brown algae taxa by dive site-group (Fig 1) and year. Pre = before dam removal.
(TIF)

S4 Fig Change over time for invertebrates and fish affected by spatially broad processes. Mean density (±SE) versus year for responses where models with x2 = year fit better than models with x2 = change in reflectance or change in algae cover (Table 5). Better fit for models with year may indicate spatially broad effects unrelated to dam removal, for example recruitment or disease (see text). The means (i.e., points) in each plot were generated by holding all predictors other than year constant at their mean (and substrate change equal to no). Density was ln(y+1)-transformed and then standardized (difference between the observation and the mean divided by the standard deviation) prior to analysis. Because of standardization, magnitudes of annual differences can be compared among plots. Before = before dam removal.
(TIF)

S1 Table Dates of surveys for secondary cover of macroalgae at dive sites. (PDF)

S2 Table Survey dates for towed underwater videography. (PDF)

S3 Table Sample size of remotely-sensed light reflectance during dam removal by month and water year. (PDF)

S4 Table Substrate grain size at dive sites. (PDF)

S5 Table Total vegetation cover at video transect segments. (PDF)

S6 Table Taxa surveyed for density and species present at dive sites. (PDF)

S7 Table Abundance of macroalgae, invertebrates, and fish at dive sites before dam removal. (PDF)

S8 Table General additive models (GAMs) of the response of vegetation cover (%) to a combination of predictors. (PDF)

S9 Table Analyses to test for response differences between the two types of substrate change. (PDF)

S1 Text Analysis methods details. (PDF)

S2 Text Limitations of BACI, diver observations, and light reflectance. (PDF)
