# Supplementary figures and images for: Increased sediment load during a large-scale dam removal changes nearshore subtidal communities
Source: PLoS One. 2017 Dec 8;12(12):e0187742. doi: 10.1371/journal.pone.0187742 (PMC5722376; doi:10.1371/journal.pone.0187742)

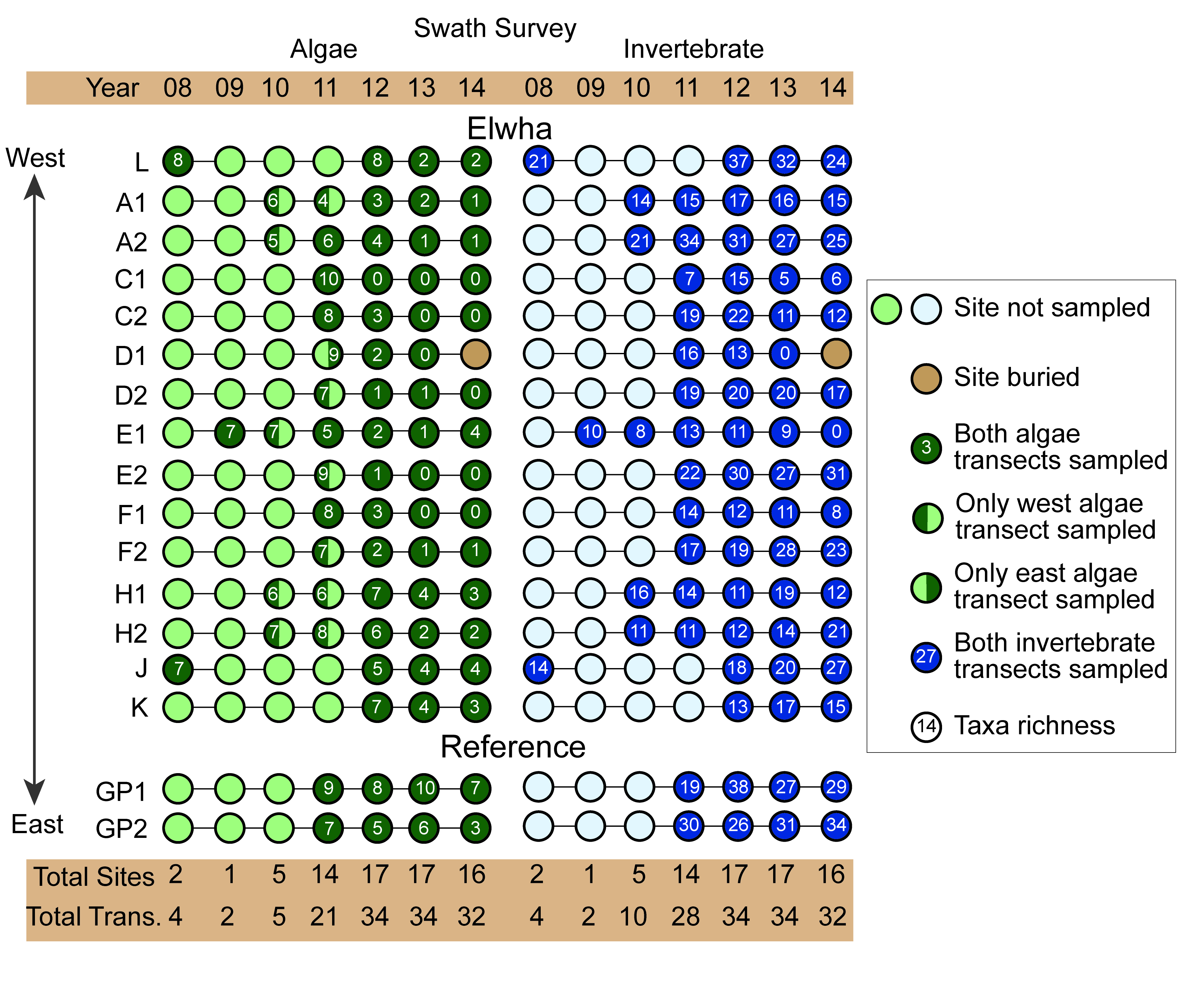

Supplement: S1 Fig — (TIF) [file pone.0187742.s001.tif]

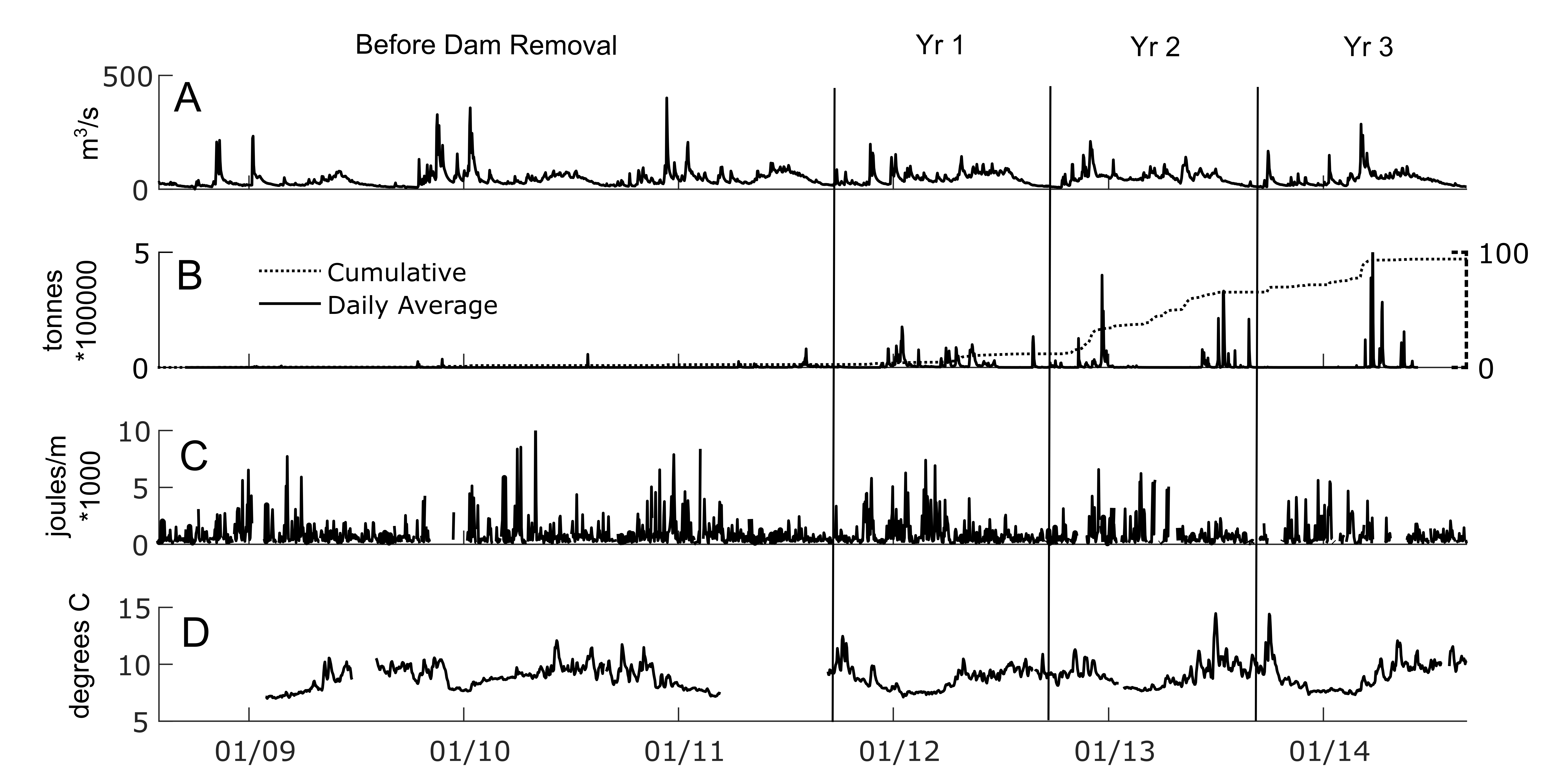

Supplement: S2 Fig — (A) Elwha River mean daily discharge and (B) sediment flux (cumulative and daily average, in tonnes) estimated at USGS River gage 12046260 (rkm 5), (C) mean daily wave energy flux at NOAA Hein Bank buoy (Fig 1), and (D) mean daily water temperature at a depth of 6m MLLW at site E1 near the Elwha River mouth. (TIF) [file pone.0187742.s002.tif]

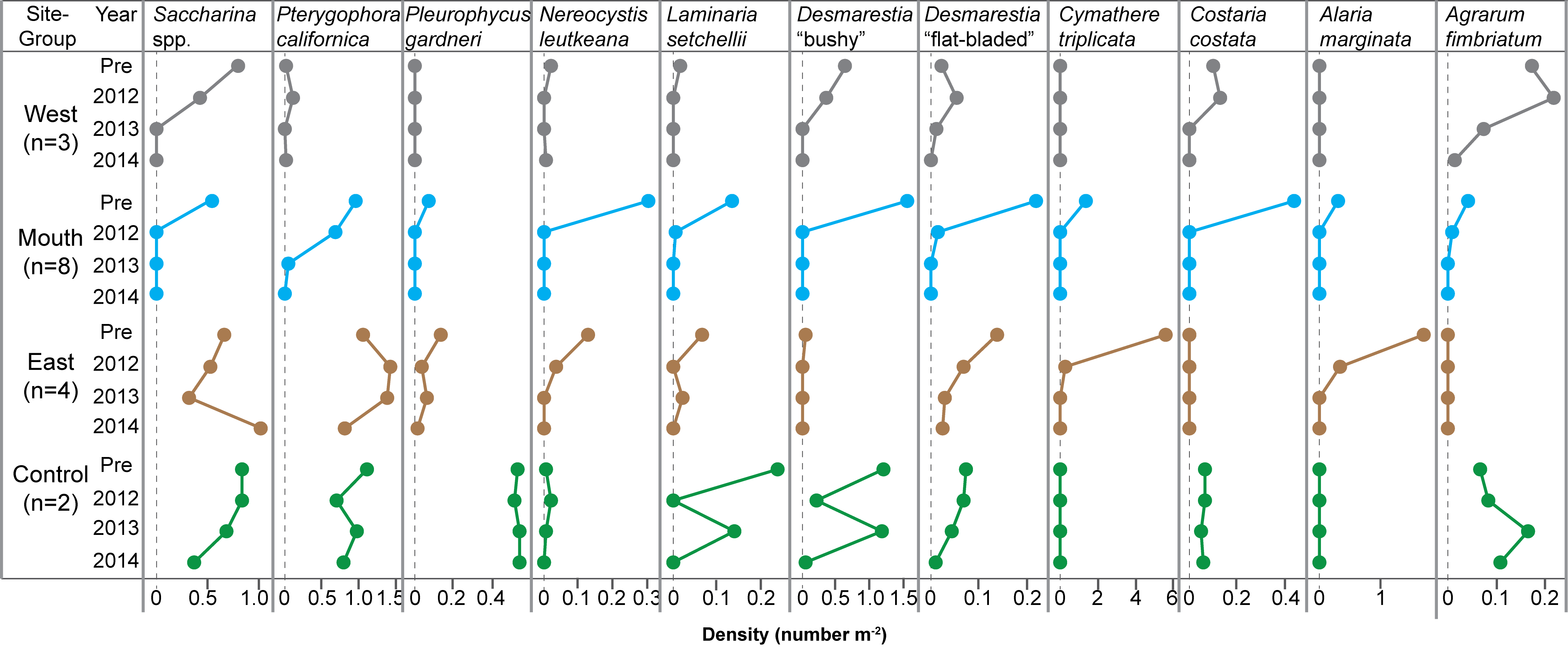

Supplement: S3 Fig — Pre = before dam removal. (TIF) [file pone.0187742.s003.tif]
