# Supplementary material for: Increased sediment load during a large-scale dam removal changes nearshore subtidal communities
Source: PLoS One. 2017 Dec 8;12(12):e0187742. doi: 10.1371/journal.pone.0187742 (PMC5722376; doi:10.1371/journal.pone.0187742)
Supplement: S1 Table — (PDF) [file pone.0187742.s005.pdf]

S1 Table. Dates of surveys for secondary cover of macroalgae at dive sites. Surveys were conducted using the Uniform Point Contact (UPC) super-layer methodology.

		Year						
Site	Transect	2008	2009	2010	2011	2012	2013	2014
A1	E			8/30	8/6	7/25	8/12	7/21
	W			8/30	8/6	7/25	8/12	7/21
A2	E			8/30	8/5	8/7	7/29	8/17
	W			8/30	8/21	7/24	7/29	7/21
C1	E				8/7	7/28	8/1	7/23
	W				8/8	7/28	8/1	7/23
C2	E				8/20	7/27	8/13	7/23
	W				8/20	7/26	8/12	7/23
D1	E				8/22	7/25	8/13	
	W				8/22	7/25	8/13	
D2	E				8/19	7/24	7/30	8/16
	W				8/19	7/24	7/30	8/16
E1	E		9/12	8/31	8/21	7/25	8/14	7/21
	W		9/12	9/1	8/21	7/25	8/13	7/21
E2	E				8/20	8/8	8/14	8/4
	W				8/20	8/8	8/13	8/4
F1	E				8/7	7/27	8/12	8/3
	W				8/7	7/27	8/12	8/3
F2	E				8/7	8/7	7/31	8/17
	W				8/20	8/7	7/31	8/17
H1	E			8/28	8/8	7/28	8/16	7/23
	W			8/28	8/21	7/28	8/12	7/23
H2	E			8/31	8/6	8/8	7/29	7/22
	W			8/31	8/6	8/8	7/29	7/22
J	E	9/8				8/21	8/16	7/22
	W	9/8				8/21	7/31	7/22
K	E					8/24	7/30	7/20
	W					8/24	7/30	7/20
L	E	9/8				8/25	8/30	7/19
	W	9/8				8/25	8/30	7/19
GP1	E				8/18	8/9	8/27	8/20
	W				8/18	8/9	8/15	8/20
GP2	E				8/18	8/9	8/27	8/2
	W				8/18	8/9	8/15	8/2
