# Supplementary material for: Increased sediment load during a large-scale dam removal changes nearshore subtidal communities
Source: PLoS One. 2017 Dec 8;12(12):e0187742. doi: 10.1371/journal.pone.0187742 (PMC5722376; doi:10.1371/journal.pone.0187742)
Supplement: S2 Table — (PDF) [file pone.0187742.s006.pdf]

S2 Table. Survey dates for towed underwater videography; tran = transect.

Geographic Area	Transect numbers	Year			
		**2010**	**2012**	**2013**	**2014**
Crescent Bay	1-10	7/23	7/30	7/8, 7/9	8/30
Freshwater Bay	11-15	7/23	7/31	7/8, 7/9	8/30
Elwha Bluffs	16-20	7/24	7/31, 8/1	7/8 (tran 19, 20)<br>8/18 (tran 16, 17, 18)	8/29
Ediz Hook	21-30	7/24	7/31	8/17	8/29
Dungeness Bluffs	31-35	7/21, 7/25	8/1, 8/2	7/10 (tran 31, 32)<br>8/16, 8/17 (tran 33, 34, 35)	8/28, 8/31
