# Supplementary material for: Increased sediment load during a large-scale dam removal changes nearshore subtidal communities
Source: PLoS One. 2017 Dec 8;12(12):e0187742. doi: 10.1371/journal.pone.0187742 (PMC5722376; doi:10.1371/journal.pone.0187742)
Supplement: S3 Table — (PDF) [file pone.0187742.s007.pdf]

S3 Table. Sample size of remotely-sensed light reflectance during dam removal by month and water year. Sample size is the number of clear days providing usable data. Mean reflectance (%) is also given.

	Water year (October-September)			
Month	2012	2013	2014	Total
10	3 (1.3)	5 (1.2)	2 (2.5)	10 (1.5)
11	1 (2.1)		1 (1.6)	2 (1.9)
12	1 (2.0)	1 (1.2)	1 (1.5)	3 (1.6)
1	2 (1.8)			2 (1.8)
2	2 (2.1)		2 (1.7)	4 (1.9)
3	2 (1.7)	4 (2.0)	1 (4.2)	7 (2.3)
4		2 (2.4)		2 (2.4)
5	4 (1.6)	3 (2.4)	3 (1.4)	10 (1.8)
6	1 (1.3)	3 (1.9)	2 (1.4)	6 (1.7)
7	6 (1.4)	5 (1.0)	4 (0.8)	15 (1.1)
8	3 (1.0)	1 (0.7)	2 (1.0)	6 (0.9)
9	8 (0.7)		8 (1.0)	16 (0.8)
Total	33 (1.3)	24 (1.6)	26 (1.4)	83 (1.4)
