# Supplementary material for: Increased sediment load during a large-scale dam removal changes nearshore subtidal communities
Source: PLoS One. 2017 Dec 8;12(12):e0187742. doi: 10.1371/journal.pone.0187742 (PMC5722376; doi:10.1371/journal.pone.0187742)
Supplement: S4 Table — (PDF) [file pone.0187742.s008.pdf]

S4 Table. Substrate grain size at dive sites. Fractional composition (by dry weight) and median grain size of physical sediment samples, and fraction of Uniform Point Contact (UPC) tallies falling into three categories (MS = Mud/Sand, GC = Gravel/Cobble, BB = Boulder/Bedrock) for sites sampled in 2012, 2013 and 2014. NS=Not Sampled, "*"= sample not analyzed, only described as "all gravel".

Site	Mud (%)	Sand (%)	Gravel (%)	Mass (g)	D50 (mm)	MS (%)	GC (%)	BB (%)
					**2012 / 2013 / 2014**			
L	NS/27/30	NS/63/68	NS/10/2	NS/18/5	NS/0.28/0.19	NS/89/83	NS/3/8	NS/8/9
A1	10/70/18	90/30/82	0/0/0	16/4/14	0.12/0.03/0.10	82/97/99	1/3/1	17/0/0
A2	3/19/9	86/52/73	11/28/18	27/28/26	0.51/0.56/0.72	93/96/100	5/2/0	2/2/0
C1	10/88/91	89/12/9	1/0/0	27/6/5	0.34/0.02/0.01	98/100/100	2/0/0	0/0/0
C2	4/88/91	81/12/9	14/0/0	35/8/5	0.58/0.02/0.01	99/100/100	1/0/0	0/0/0
D1	1/2/NS	46/93/NS	53/5/NS	61/38/NS	1.69/0.54/NS	26/61/NS	72/39/NS	2/0/NS
D2	3/6/3	46/46/49	51/48/48	42/43/42	1.91/1.83/1.78	18/4/11	82/96/88	0/0/0
E1	0/4/3	38/70/97	61/26/1	69/33/17	1.60/0.34/0.41	6/98/100	93/1/0	1/1/0
E2	4/7/10	84/54/77	12/39/14	37/32/24	0.63/0.91/0.61	6/1/3	86/92/93	7/8/5
F1	1/1/5	22/5/92	77/95/3	110/80/19	5.96/7.43/0.49	11/22/94	86/77/6	3/1/0
F2	0/5/5	0/28/29	100/67/66	*/55/68	*/4.92/5.26	0/5/4	84/93/96	16/2/0
H1	2/2/2	28/31/35	69/67/84	75/59/74	4.79/4.18/6.61	5/9/7	92/91/92	3/0/1
H2	2/3/4	57/32/35	41/66/61	50/66/59	1.56/4/40/4.32	2/6/1	96/93/98	2/1/1
J	NS/2/3	NS/29/44	NS/69/54	NS/51/51	NS/5.34/2.00	NS/60/43	NS/38/57	NS/1/0
K	NS/2/5	NS/19/33	NS/78/62	NS/54/43	NS/6.16/4.65	NS/25/17	NS/75/83	NS/0/0
GP1	NS/2/5	NS/38/67	NS/60/29	NS/53/24	NS/1.54/0.92	NS/37/45	NS/52/45	NS/10/10
GP2	NS/5/3	NS/86/90	NS/9/7	NS/38/20	NS/0.39/0.43	NS/73/61	NS/25/37	NS/2/2
