# Supplementary material for: Increased sediment load during a large-scale dam removal changes nearshore subtidal communities
Source: PLoS One. 2017 Dec 8;12(12):e0187742. doi: 10.1371/journal.pone.0187742 (PMC5722376; doi:10.1371/journal.pone.0187742)
Supplement: S5 Table — (PDF) [file pone.0187742.s009.pdf]

S5 Table. Total vegetation cover at video transect segments. Entries are annual mean percent cover and standard deviation. Initial number of samples and initial depth (i.e., from the 2010 survey) are included. Each transect was subdivided into 200 m sections and labelled alphabetically from shallow to deep.

Transect (segment)	Initial samples (n)	Initial depth (m)	2010 cover mean (SD)	2012 cover mean (SD)	2013 cover mean (SD)	2014 cover mean (SD)
1(a)	31	2.7	50 (40)	80 (24)	85 (19)	91 (4)
1(b)	31	6.9	73 (25)	67 (26)	72 (26)	65 (27)
1(c)	35	10.2	31 (19)	39 (15)	41 (17)	43 (13)
1(d)	39	14.3	37 (20)	44 (26)	30 (25)	20 (19)
1(e)	52	10.2	70 (25)	74 (23)	69 (31)	52 (32)
2(a)	35	2.8	38 (40)	66 (34)	76 (29)	90 (9)
2(b)	33	7.1	69 (23)	73 (23)	86 (12)	74 (19)
2(c)	33	10.0	31 (19)	42 (16)	42 (19)	37 (15)
2(d)	45	13.8	35 (17)	49 (17)	33 (19)	24 (18)
2(e)	51	11.7	55 (25)	59 (26)	53 (27)	34 (28)
3(a)	33	3.4	34 (34)	52 (39)	54 (37)	78 (23)
3(b)	35	7.3	48 (32)	63 (31)	43 (27)	60 (30)
3(c)	33	10.0	41 (19)	32 (15)	36 (21)	35 (15)
3(d)	34	14.5	39 (14)	42 (15)	35 (14)	22 (12)
3(e)	64	14.0	40 (28)	42 (25)	40 (29)	23 (21)
4(a)	39	5.4	0 (2)	5 (8)	5 (17)	20 (20)
4(b)	38	7.9	1 (8)	7 (4)	6 (9)	16 (14)
4(c)	37	10.4	14 (12)	15 (11)	23 (17)	23 (14)
4(d)	55	15.3	30 (23)	28 (17)	41 (22)	17 (14)
5(a)	38	5.3	0 (0)	4 (4)	1 (2)	17 (17)
5(b)	37	7.6	1 (3)	8 (8)	3 (6)	13 (9)
5(c)	40	9.7	15 (14)	17 (13)	22 (21)	22 (13)
5(d)	38	15.1	35 (22)	17 (14)	21 (19)	19 (13)
6(a)	42	4.1	6 (17)	7 (17)	4 (15)	11 (15)
6(b)	41	7.5	8 (8)	12 (18)	7 (18)	12 (9)
6(c)	46	12.1	15 (11)	27 (17)	30 (19)	19 (14)
7(a)	40	4.5	7 (21)	4 (9)	1 (4)	14 (18)
7(b)	43	8.1	8 (9)	8 (9)	8 (10)	12 (9)
7(c)	70	12.9	31 (22)	46 (33)	34 (25)	34 (25)
8(a)	45	5.6	1 (4)	7 (10)	4 (13)	10 (9)
8(b)	61	10.2	26 (23)	29 (26)	19 (25)	25 (20)
8(c)	67	7.7	92 (0)	81 (19)	90 (10)	83 (16)
9(a)	34	4.8	5 (16)	5 (10)	6 (17)	12 (15)
9(b)	32	7.7	16 (15)	8 (4)	8 (13)	13 (11)
9(c)	34	9.3	55 (28)	53 (28)	51 (28)	40 (27)
9(d)	27	9.7	77 (25)	76 (25)	67 (32)	66 (33)
10(a)	51	6.6	14 (32)	13 (26)	4 (10)	10 (11)

10(b)	49	7.7	79 (31)	74 (24)	83 (20)	74 (19)
10(c)	38	12.9	50 (32)	54 (24)	43 (29)	27 (25)
11(a)	28	1.6	43 (38)	73 (30)	72 (29)	88 (11)
11(b)	30	5.1	57 (29)	51 (35)	36 (37)	54 (40)
11(c)	29	11.1	57 (13)	23 (14)	7 (8)	8 (5)
11(d)	26	16.1	73 (13)	12 (11)	2 (3)	4 (4)
12(a)	45	14.8	60 (16)	38 (21)	12 (16)	2 (3)
12(b)	39	16.9	50 (19)	30 (25)	0 (3)	5 (4)
13(a)	38	13.8	75 (16)	39 (23)	16 (17)	13 (16)
13(b)	17	16.2	64 (19)	24 (23)	1 (2)	5 (4)
14(a)	40	5.4	29 (35)	12 (24)	13 (26)	13 (20)
14(b)	41	10.3	51 (27)	7 (10)	2 (3)	15 (9)
14(c)	48	12.9	46 (15)	8 (11)	2 (4)	7 (8)
14(d)	54	14.4	52 (17)	20 (24)	4 (11)	4 (5)
14(e)	56	15.8	50 (16)	17 (21)	1 (4)	1 (4)
14(f)	22	17.4	54 (9)	27 (20)	4 (8)	1 (3)
15(a)	37	6.1	77 (20)	1 (2)	0 (0)	0 (0)
15(b)	51	10.2	59 (14)	7 (4)	0 (0)	3 (4)
15(c)	57	14.6	47 (19)	5 (5)	0 (0)	6 (5)
16(a)	39	6.5	66 (21)	6 (6)	5 (5)	28 (34)
16(b)	46	9.5	73 (15)	5 (4)	8 (3)	51 (17)
16(c)	40	11.5	87 (7)	8 (0)	10 (5)	38 (13)
16(d)	42	12.7	68 (14)	10 (7)	7 (2)	24 (8)
16(e)	44	13.8	70 (13)	18 (13)	9 (4)	27 (13)
16(f)	58	15.4	65 (14)	11 (10)	7 (3)	15 (9)
17(a)	34	4.9	88 (11)	55 (18)	48 (23)	77 (19)
17(b)	33	7.0	66 (15)	26 (15)	9 (22)	66 (17)
17(c)	35	8.2	71 (20)	23 (17)	21 (14)	71 (15)
17(d)	40	9.3	73 (19)	21 (16)	21 (8)	67 (17)
17(e)	39	10.0	59 (20)	18 (12)	22 (10)	62 (18)
17(f)	43	11.6	49 (15)	14 (8)	10 (6)	66 (16)
17(g)	46	13.0	49 (23)	12 (9)	7 (3)	49 (11)
17(h)	42	13.7	42 (16)	9 (3)	8 (0)	42 (13)
17(i)	52	15.7	30 (18)	7 (4)	4 (4)	21 (11)
18(a)	39	4.4	87 (11)	78 (19)	69 (20)	87 (10)
18(b)	37	5.8	57 (15)	22 (15)	42 (18)	53 (21)
18(c)	37	6.5	47 (19)	25 (16)	47 (15)	45 (15)
18(d)	40	7.0	33 (17)	20 (15)	49 (8)	26 (18)
18(e)	41	8.6	17 (11)	9 (3)	21 (10)	27 (15)
18(f)	32	9.3	28 (19)	20 (17)	9 (3)	16 (11)
18(g)	42	10.0	60 (18)	30 (18)	18 (12)	42 (12)
18(h)	37	10.9	62 (16)	40 (13)	35 (13)	43 (16)
18(i)	38	11.7	62 (18)	41 (14)	29 (10)	53 (10)

18(j)	36	13.0	63 (16)	30 (15)	10 (5)	51 (12)
18(k)	41	14.8	54 (12)	21 (16)	6 (3)	41 (15)
19(a)	32	6.4	22 (29)	1 (3)	1 (2)	8 (10)
19(b)	32	9.5	61 (20)	20 (17)	9 (11)	32 (16)
19(c)	36	10.6	45 (31)	22 (19)	3 (5)	18 (13)
19(d)	35	11.6	44 (20)	25 (18)	3 (4)	10 (6)
19(e)	39	12.7	51 (25)	28 (17)	4 (4)	11 (8)
19(f)	43	14.1	72 (14)	44 (12)	8 (2)	33 (16)
19(g)	34	16.3	73 (13)	42 (12)	10 (5)	10 (10)
20(a)	32	6.0	71 (33)	52 (34)	50 (38)	59 (38)
20(b)	34	9.9	61 (21)	31 (18)	14 (11)	31 (19)
20(c)	34	11.6	70 (17)	30 (21)	11 (8)	19 (13)
20(d)	35	12.2	67 (15)	35 (17)	12 (7)	21 (11)
20(e)	39	12.6	49 (28)	24 (18)	7 (4)	9 (4)
20(f)	38	14.1	78 (14)	47 (9)	9 (3)	12 (7)
20(g)	42	16.4	33 (20)	20 (17)	5 (4)	11 (9)
21(a)	36	5.9	38 (33)	27 (25)	28 (21)	28 (23)
21(b)	33	7.5	35 (14)	33 (17)	46 (13)	16 (11)
21(c)	35	8.6	31 (22)	28 (18)	20 (15)	9 (13)
21(d)	38	12.1	60 (19)	38 (23)	29 (23)	22 (22)
22(a)	38	5.7	34 (21)	32 (19)	56 (27)	20 (19)
22(b)	33	7.7	32 (17)	29 (18)	34 (19)	15 (16)
22(c)	42	12.4	50 (16)	34 (25)	15 (14)	11 (11)
23(a)	40	5.2	45 (28)	50 (31)	48 (32)	46 (32)
23(b)	31	11.0	56 (26)	33 (23)	31 (26)	28 (25)
24(a)	19	5.4	74 (24)	43 (32)	54 (28)	57 (25)
25(a)	33	7.4	52 (36)	33 (30)	41 (32)	37 (28)
26(a)	29	8.5	61 (33)	45 (32)	52 (29)	49 (25)
27(a)	35	7.8	61 (31)	39 (29)	44 (31)	42 (29)
28(a)	17	9.8	59 (30)	48 (32)	45 (34)	34 (29)
29(a)	11	9.2	71 (28)	55 (38)	52 (38)	58 (39)
30(a)	10	7.2	87 (8)	74 (20)	68 (32)	72 (29)
31(a)	39	5.5	31 (20)	42 (24)	35 (28)	37 (25)
31(b)	41	6.8	64 (29)	70 (28)	55 (35)	60 (27)
31(c)	42	8.3	61 (22)	66 (25)	47 (27)	48 (24)
31(d)	39	9.3	62 (18)	55 (26)	52 (30)	34 (21)
31(e)	37	10.2	65 (19)	66 (19)	56 (23)	29 (16)
31(f)	42	12.6	52 (9)	61 (13)	40 (16)	26 (12)
31(g)	42	14.2	56 (14)	51 (15)	31 (14)	10 (6)
31(h)	45	14.4	59 (13)	39 (16)	35 (15)	6 (3)
31(i)	52	15.1	56 (15)	25 (17)	14 (16)	5 (4)
32(a)	43	5.0	48 (27)	59 (35)	63 (29)	55 (40)
32(b)	45	7.4	57 (21)	70 (23)	58 (30)	58 (27)

32(c)	45	8.7	27 (14)	45 (23)	35 (26)	25 (20)
32(d)	42	9.3	35 (14)	50 (17)	25 (20)	22 (16)
32(e)	42	9.4	33 (14)	38 (15)	14 (14)	20 (12)
32(f)	40	10.3	49 (12)	46 (17)	28 (19)	34 (19)
32(g)	45	13.6	53 (15)	46 (18)	32 (16)	27 (13)
33(a)	18	5.5	89 (10)	88 (8)	86 (12)	88 (10)
33(b)	29	6.9	66 (19)	82 (16)	70 (20)	57 (27)
33(c)	34	9.3	64 (15)	72 (17)	59 (18)	52 (20)
33(d)	38	12.7	47 (18)	40 (24)	19 (14)	28 (21)
33(e)	45	16.1	27 (15)	15 (16)	7 (5)	9 (9)
33(f)	7	18.1	8 (0)	3 (4)	2 (3)	1 (2)
34(a)	38	5.4	12 (22)	11 (15)	9 (19)	8 (21)
34(b)	33	8.2	19 (14)	18 (12)	11 (8)	17 (16)
34(c)	36	10.8	23 (9)	15 (8)	13 (9)	15 (12)
34(d)	37	13.1	23 (11)	15 (9)	8 (3)	9 (5)
34(e)	38	14.4	24 (13)	10 (5)	7 (2)	8 (4)
34(f)	42	15.3	40 (13)	17 (8)	10 (6)	9 (5)
34(g)	29	16.5	36 (13)	17 (8)	10 (5)	14 (8)
35(a)	31	5.2	87 (11)	77 (20)	90 (9)	73 (22)
35(b)	30	7.5	66 (29)	54 (27)	57 (28)	39 (19)
35(c)	32	9.3	53 (22)	45 (26)	39 (26)	25 (18)
35(d)	29	10.5	44 (11)	37 (20)	24 (22)	16 (10)
35(e)	32	11.6	47 (13)	27 (17)	20 (18)	18 (14)
35(f)	33	12.4	38 (13)	33 (16)	18 (13)	13 (8)
35(g)	36	13.5	32 (12)	27 (18)	13 (10)	12 (9)
35(h)	37	14.2	20 (9)	24 (16)	11 (7)	13 (9)
35(i)	39	14.4	22 (18)	19 (19)	19 (17)	18 (21)
35(j)	37	14.9	35 (15)	40 (13)	23 (13)	26 (14)
35(k)	34	15.7	43 (12)	39 (13)	16 (8)	17 (8)
