# Supplementary material for: Increased sediment load during a large-scale dam removal changes nearshore subtidal communities
Source: PLoS One. 2017 Dec 8;12(12):e0187742. doi: 10.1371/journal.pone.0187742 (PMC5722376; doi:10.1371/journal.pone.0187742)
Supplement: S6 Table — (PDF) [file pone.0187742.s010.pdf]

S6 Table. Taxa surveyed for density and species present at dive sites. Fine analysis group = lowest practicable taxonomic level for consistent identification. Coarse analysis group = grouping used in some invertebrate and fish analyses (see text). Species present = species present during our surveys, often identified from photos. The Species Present column only includes species that we positively identified; additional species that we were unable to identify were also present.

Group	Phylum or class	Taxonomic level 2	Coarse analysis group	Fine analysis group	Species present
Macroalgae	Phaeophyceae (Brown algae)	Desmarestia (acid kelp)		*Desmarestia* bushy	*Desmarestia aculeata*
					Desmarestia viridis
				Desmarestia flat-bladed	*Desmarestia foliacea*
					Desmarestia ligulata
					Desmarestia latissima
		Laminariales (kelp)		*Agarum fimbriatum*	*Agarum fimbriatum*
				Alaria marginata	*Alaria marginata*
				Costaria costata	*Costaria costata*
				Cymathere triplicata	*Cymathere triplicata*
				Laminaria ephemera	*Laminaria ephemera*
				Laminaria setchellii	*Laminaria setchellii*
				Nereocystis luetkeana	*Nereocystis luetkeana*
				Pleurophycus gardneri	*Pleurophycus gardneri*
				Pterygophora californica	*Pterygophora californica*
				Saccharina spp.	*Saccharina groenlandica*
					Saccharina latissima
Invertebrates	Annelida	Polychaete	Tube worm	*Bispira* spp.	*Bispira* spp.
				Chone aurantiaca	*Chone aurantiaca*
				Diopatra ornata	*Diopatra ornata*
				Eudistylia polymorpha	*Eudistylia polymorpha*
				Eudistylia vancouveri	*Eudistylia vancouveri*
				Myxicola spp.	*Myxicola infundibulum*
					Myxicola spp.
				Orange feather duster	Unknown
				Pista pacifica	*Pista pacifica*

			Schizobranchia spp.	*Schizobranchia* spp.
			Spaghetti worm	Unknown
			Tube worm other	*Demonax medius*
				Phyllochaetopterus claparedii
				Phyllochaetopterus prolifica
				Soft – unknown
Arthropoda	Barnacle	Barnacle	*Balanus nubilus*	*Balanus nubilus*
	Decapod	Crab other	Crab other	Unknown
			Lithodidae	*Cryptolithodes sitchensis*
				Cryptolithodes typicus
				Lopholithodes mandtii
				Phyllolithodes papillosus
				Rhinolithodes wosnessenskii
			Telmessus cheiragonus	*Telmessus cheiragonus*
	Hermit crab (Paguridae)	Hermit crab	*Elassochirus tenuimanus*	*Elassochirus tenuimanus*
			Hermit other	*Pagurus ochotensis*
				Pagurus spp.
				Unknown
			Pagurus armatus	*Pagurus armatus*
			Pagurus beringanus	*Pagurus beringanus*
			Pagurus kennerlyi	*Pagurus kennerlyi*
			Sponge hermit crab	*Pagurus stevensae*
				Unknown
	Shrimp (Caridea)	Shrimp	Crangon spp.	*Metacrangon munita*
				Crangon spp.
			Heptacarpus spp.	*Heptacarpus stylus*
				Heptacarpus spp.
			Pandalus spp.	*Pandalus danae*
				Pandalus spp.

			Shrimp other	Unknown
	True crab (Brachyura)	Cancer crab	*Cancer gracilis*	*Cancer gracilis*
			Cancer oregonensis	*Cancer oregonensis*
			Cancer productus	*Cancer productus*
			Metacarcinus magister	*Metacarcinus magister*
			Metacarcinus magister juvenile	*Metacarcinus magister*
		Spider crab (Majoidea)	*Oregonia gracilis*	*Oregonia gracilis*
			Pugettia gracilis	*Pugettia gracilis*
			Pugettia producta	*Pugettia producta*
			Scyra acutifrons	*Scyra acutifrons*
			Spider crab other	Spider crab - unknown
Brachiopoda	Brachiopod	Brachiopod	*Terebratalia transversa*	*Terebratalia transversa*
Chordata	Tunicate	Tunicate	Solitary tunicate other	*Boltenia villosa*
				Cnemidocarpa finmarkiensis
				Pyura haustor
			Styela montereyensis	*Styela montereyensis*
Cnidaria	Anemone	Anemone Halcampa	*Halcampa* spp.	*Halcampa crypta*
				Halcampa decemtentaculata
		Anemone other	Anemone other	*Metridium senile*
				Pachycerianthus fimbriatus
				Large white - unknown
				Peachia quinquecapitata
			Epiactis spp.	*Epiactis lisbethae*
				Epiactis prolifera
				Epiactis ritteri
			Stomphia spp.	*Stomphia coccinea*

				Stomphia didemon
			Urticina columbiana	*Urticina columbiana*
			Urticina coriacea	*Urticina coriacea*
			Urticina crassicornis	*Urticina crassicornis*
			Urticina lofotensis	*Urticina lofotensis*
			Urticina piscivora	*Urticina piscivora*
	Jelly	Stalked jelly	*Haliclystus* spp.	*Haliclystus* spp.
	Sea pen	Sea pen	*Ptilosarcus gurneyi*	*Ptilosarcus gurneyi*
Echinodermata	Sea cucumber	Sea cucumber	*Cucumaria miniata*	*Cucumaria miniata*
			Cucumber other	*Cucumaria piperata*
				Eupentacta quinquesemita
				Psolus spp.
				White - unknown
			Parastichopus californicus	*Parastichopus californicus*
	Sea star	Sea star	*Dermasterias imbricata*	*Dermasterias imbricata*
			Evasterias troschelii	*Evasterias troschelii*
			Henricia spp.	*Henricia leviuscula leviuscula*
				Henricia sanguinolenta
				Henricia pumila
			Luidia foliolata	*Luidia foliolata*
			Mediaster aequalis	*Mediaster aequalis*
			Orthasterias koehleri	*Orthasterias koehleri*
			Pisaster brevispinus	*Pisaster brevispinus*
			Pteraster tesselatus	*Pteraster tesselatus*
			Pycnopodia helianthoides	*Pycnopodia helianthoides*
			Solaster spp.	*Solaster dawsoni*
				Solaster endeca
				Solaster stimpsoni

			Star other	Unknown
	Sea urchin	Sea urchin	*Strongylocentrotus droebachiensis*	*Strongylocentrotus droebachiensis*
			Strongylocentrotus franciscanus	*Strongylocentrotus franciscanus*
Mollusca	Bivalve	Bivalve	Bivalve other	*Penitella* spp.
				Platyodon cancellatus
				Pododesmus macrochisma
				Unknown
			Chlamys spp.	*Chlamys* spp.
			Clinocardium nuttallii	*Clinocardium nuttallii*
			Crassedoma gigantea	*Crassedoma gigantea*
			Humilaria kennerleyi	*Humilaria kennerleyi*
			Mya truncata	*Mya truncata*
			Panomya ampla	*Panomya ampla*
			Panopea generosa	*Panopea generosa*
			Saxidomus gigantea	*Saxidomus gigantea*
			Tresus capax	*Tresus capax*
			Zirfaea pilsbryi	*Zirfaea pilsbryi*
	Octopus	Octopus	*Enteroctopus dofleini*	*Enteroctopus dofleini*
			Octopus rubescens	*Octopus rubescens*
	Chiton	Chiton	*Cryptochiton stelleri*	*Cryptochiton stelleri*
			Katharina tunicata	*Katharina tunicata*
			Lepidozona spp.	*Lepidozona mertensii*
				Lepidozona spp.
			Mopalia spp.	*Mopalia kennerleyi*
				Mopalia lignosa
			Tonicella spp.	*Tonicella insignis*
				Tonicella lineata
				Tonicella undocaerulea

			Tonicella venusta
			Tonicella spp.
Gastropod	Limpet	*Acmaea mitra*	*Acmaea mitra*
		Diodora aspera	*Diodora aspera*
		Limpet other	*Tectura scutum*
			Unknown
		Lottia instabilis	*Lottia instabilis*
	Nudibranch	Aeolidida	Aeolidia papillosa
			Cuthona spp.
			Flabellina trilineata
			Flabellina trophina
			Flabellina verrucosa
			Flabellina spp.
			Hermissenda crassicornis
		Dendronotus spp.	*Dendronotus albopunctatus*
			Dendronotus albus
			Dendronotus venustus
			Dendronotus spp.
		Dirona albolineata	*Dirona albolineata*
		Doridacea	*Acanthodoris hudsoni*
			Acanthodoris nanaimoensis
			Archidoris montereyensis
			Archidoris odhneri
			Cadlina luteomarginata
			Diaulula sandiegensis
			Geitodoris heathi
			Limacia cockerelli
			Peltodoris nobilis
			Rostanga pulchra

				Nudibranch other	*Armina californica*
					Janolus fuscus
					Unknown
				Triopha catalinae	*Triopha catalinae*
			Snail	*Amphissa* spp.	*Amphissa* spp.
				Calliostoma spp.	*Calliostoma ligatum*
					Calliostoma spp.
				Ceratostoma foliatum	*Ceratostoma foliatum*
				Euspira lewisii	*Euspira lewisii*
					Euspira lewisii egg case
				Fusitriton oregonensis	*Fusitriton oregonensis*
				Snail other	*Nassarius fossatus*
					Calyptraea fastigiata
					Gastropteron pacificum
					Nucella lamellosa
					Nucella spp.
					Olivella (*Callianax*) *baetica*
					Trichotropis cancellata
					Unknown
	Nemertea	Nemertea	Ribbon worm	*Tubulanus polymorphus*	*Tubulanus polymorphus*
	Porifera	Sponge	Sponge	Solitary sponge	*Clathrina* spp.
					Cliona celata californiana
					Leucilla nuttingi
					Stylissa stipitata
					Suberites domuncula
					Solitary sponge unknown
Fish	Chondrichthyes (cartilaginous fishes)	Chimaeridae	Ratfish	Spotted ratfish	*Hydrolagus colliei*
		Squalidae	Dogfish	Spiny dogfish	*Squalus suckleyi*

Osteichthyes (bony fishes)	Agonidae	Poacher	Poacher	*Agonopsis vulsa*
				Bothragonus swanii
				Anoplagonus inermis
				Podothecus accipenserinus
	Ammodytidae	Sand lance	Pacific sand lance	*Ammodytes hexapterus*
	Clupeidae	Herring	Pacific herring	*Clupea pallasii pallasii*
	Cottoidei	Sculpin	Buffalo sculpin	*Enophrys bison*
			Great sculpin	*Myoxocephalus polyacanthocephalus*
			Longfin sculpin	*Jordania zonope*
			Manacled sculpin	*Synchirus gilli*
			Red Irish lord	*Hemilepidotus hemilepidotus*
			Scalyhead sculpin	*Artedius harringtoni*
			Sculpin other	*Scorpaenichthys marmoratus*
				Rhamphocottus richardsonii
				Nautichthys oculofasciatus
				Unknown
			Silverspotted sculpin	*Blepsias cirrhosus*
	Embiotocidae	Perch	Perch	*Rhacochilus vacca*
				Cymatogaster aggregata
				Embiotoca lateralis
				Unknown
	Gadidae	Cod	Pacific cod	*Gadus macrocephalus*
	Gobiidae	Goby	Blackeye goby	*Rhinogobiops nicholsii*
	Hexagrammidae	Greenling	Greenling	*Hexagrammos decagrammus*
				Hexagrammos stelleri

			Unknown
		Lingcod	*Ophiodon elongatus*
Osteichthyes	Fish other	Fish other	*Aulorhynchus flavidus*
			Engraulis mordax
			Eumicrotremus orbis
			Gasterosteus aculeatus
			Liparis greeni
			Liparis dennyi
			Snailfish - unknown
			Raja binoculata
			Skate - unknown
			Unknown
Pholidae	Gunnel	Gunnel	*Apodichthys flavidus*
			Pholis laeta
			Pholis clemensi
			Pholis schultzi
			Pholis ornata
Pleuronectidae	Flatfish	Starry flounder	*Platichthys stellatus*
Pleuronectiformes	Flatfish	Flatfish other	*Citharichthys* spp.
			Hippoglossus stenolepis
			Parophrys vetulus
			Pleuronichthys coenosus
			Psettichthys melanostictus
			Left-eye flatfish
			Right-eye flatfish
Sebastes	Rockfish	Black rockfish	*Sebastes melanops*
		Copper rockfish	*Sebastes caurinus*
		Rockfish juvenile	Unknown
		Rockfish other	*Sebastes miniatus*
			Sebastes maliger

	Stichaeidae	Prickleback	Mosshead warbonnet	*Chirolophis nugator*
			Prickleback other	*Anoplarchus insignis*
				Anoplarchus purpurescens
				Chirolophis decoratus
				Lumpenus sagitta
