# Supplementary material for: Increased sediment load during a large-scale dam removal changes nearshore subtidal communities
Source: PLoS One. 2017 Dec 8;12(12):e0187742. doi: 10.1371/journal.pone.0187742 (PMC5722376; doi:10.1371/journal.pone.0187742)
Supplement: S7 Table — (PDF) [file pone.0187742.s011.pdf]

S7 Table. Abundance of macroalgae, invertebrates, and fish at dive sites before dam removal. Entries are means averaged over all dive sites. Invertebrate and fish taxa for response = density are coarse analysis taxa from S6 Table.

Group	Response	Taxon	Mean
Macroalgae	Brown algae density ($N \cdot m^{-2}$)	*Agarum fimbriatum*	0.06
		Alaria marginata	0.49
		Costaria costata	0.25
		Cymathere triplicata	1.76
		Desmarestia bushy	0.15
		Desmarestia flat-bladed	1.06
		Laminaria ephemera	0.00
		Laminaria setchellii	0.12
		Nereocystis luetkeana	0.18
		Pleurophycus gardneri	0.14
		Pterygophora californica	0.84
		Saccharina spp.	0.66
		Total	5.69
	Fleshy red algae presence/absence index	Branch	0.643
		Bushy	0.423
		Lacy	0.226
		Leaf	0.792
		Mean	0.521
	Secondary cover (%)	Brown	39.2
		Drift	0.7
		Green	0.1
		Red	16.7
		Combination	7.4
		Total	64.0
	Primary cover (%)	Articulated coralline	0.1
		Crustose coraline	2.7
		Encrusting red	16.8
		Small fleshy red	14.0
		Live kelp holdfast	1.7
		Turf	3.4
		Total	38.8
Invertebrates	Density ($N \cdot m^{-2}$)	Anemone Halcampa	0.214
		Anemone other	0.026
		Barnacle	0.000
		Bivalve	0.165
		Brachiopod	0.001
		Cancer crab	0.018
		Chiton	0.078
		Crab other	0.019

		Hermit crab	0.054
		Limpet	0.011
		Nudibranch	0.011
		Octopus	0.000
		Ribbon worm	0.000
		Sea cucumber	0.027
		Sea pen	0.004
		Sea star	0.054
		Sea urchin	0.001
		Shrimp	0.077
		Snail	0.042
		Spider crab	0.050
		Sponge	0.003
		Stalked jelly	0.018
		Tube worm	1.763
		Tunicate	0.015
		Total	2.652
	Primary cover (%)	Barnacle	0.00
		Bivalve	0.03
		Byrozoan	1.87
		Chiton/limpet	0.16
		Cnidarian	0.00
		Echinoderm	0.11
		Hydroid	0.00
		Sponge	0.16
		Tube worm	1.36
		Tunicate	0.16
		Total	3.83
Fish	Density ($N \cdot m^{-2}$)	Cod	0.001
		Dogfish	0.002
		Fish other	0.002
		Flatfish	0.013
		Goby	0.000
		Greenling	0.010
		Gunnel	0.018
		Herring	0.209
		Perch	0.000
		Poacher	0.002
		Prickleback	0.000
		Ratfish	0.020
		Rockfish	0.001
		Sand lance	0.052
		Sculpin	0.028

Total	0.358
