# Supplementary material for: Increased sediment load during a large-scale dam removal changes nearshore subtidal communities
Source: PLoS One. 2017 Dec 8;12(12):e0187742. doi: 10.1371/journal.pone.0187742 (PMC5722376; doi:10.1371/journal.pone.0187742)
Supplement: S8 Table — (PDF) [file pone.0187742.s012.pdf]

S8 Table. General additive models (GAMs) of the response of vegetation cover (%) to a combination of predictors.

Predictor	Term type	Statistic	Dataset	
			Dive	Video
Initial depth	Smooth, ti	EDF	1.0	3.2
		F	8.9	7.4
		P	0.004	0.000
Initial percent sand	Smooth, s	EDF	4.6	NA
		F	2.7	NA
		P	0.021	NA
Reflectance change	Smooth, ti	EDF	3.3	2.3
		F	69.8	11.0
		P	0.000	0.000
Initial depth*reflectance change	Smooth, ti	EDF	2.8	1.8
		F	6.0	1.9
		P	0.001	0.100[a]
Substrate change	Parametric	Coefficient	-1.58[b]	NA
		F	41.4	NA
		P	0.000	NA
Combined		R^2_{adj}	88.7	27.2

Smooth terms: s = 1-dimensional smooth appropriate for a single predictor; ti = 2-dimensional smooth appropriate for a main effects-plus-interaction structure. EDF = estimated degrees of freedom; the greater the EDF the more wiggly the curve with EDF = 1.0 indicating a straight line. Note that initial reflectance was dropped from the dive GAM due to non-significance and was excluded from the video GAM due to high correlation with initial depth (r = -0.52).

[a]Although not significant, dropping initial depth*reflectance change from the model increased AIC_c from 1681 to 1689. We therefore judged the model that included initial depth*reflectance change to be the better model; [b]Indicates the change in ln(y+1) transposed vegetation cover for substrate change = yes relative to substrate change = no.
