# Supplementary material for: Increased sediment load during a large-scale dam removal changes nearshore subtidal communities
Source: PLoS One. 2017 Dec 8;12(12):e0187742. doi: 10.1371/journal.pone.0187742 (PMC5722376; doi:10.1371/journal.pone.0187742)
Supplement: S9 Table — (PDF) [file pone.0187742.s013.pdf]

S9 Table. Analyses to test for response differences between the two types of substrate change.

Group	Response	*P*-value		
		Initial substrate (IS)	Substrate change (SC)	IS*SC
Macroalgae	Brown algae density, assemblage multivariate similarity	0.038	0.002	0.248
Invertebrates	Density, coarser taxonomic grouping, assemblage multivariate similarity	0.039	0.028	0.062
	Bivalve density[a]	0.131	0.174	0.009
Fish	Density, coarser taxonomic grouping, assemblage multivariate similarity	0.002	0.003	0.001
	Flatfish density[a]	0.007	0.471	0.000
	Sand lance density[a]	0.003	0.003	0.002

Initial substrate types were gravel (3 sites) and sand (2 sites) (Fig 3, main paper). Substrate change consisted of sand deposition where substrate was initially gravel and mud deposition where substrate was initially sand (Fig 3, main paper). Initial substrate, substrate change (before or after), and their interaction were fixed effects; site nested within initial substrate was a random effect. A significant interaction indicates a response difference between deposition of sand on gravel and deposition of mud on sand. Multivariate tests were conducted for responses designated as "assemblage multivariate similarity"; univariate tests were conducted otherwise.

[a]Ln(y+1) transformed.
