# Supplementary material for: Increased sediment load during a large-scale dam removal changes nearshore subtidal communities
Source: PLoS One. 2017 Dec 8;12(12):e0187742. doi: 10.1371/journal.pone.0187742 (PMC5722376; doi:10.1371/journal.pone.0187742)
Supplement: S1 Text — (PDF) [file pone.0187742.s014.pdf]

S1 Text: Analysis methods details

Table 1 in the main body of the paper lists three main questions of concern and summarizes analyses used to address them. In the main body of the paper we describe the major features of the analyses to give readers an understanding our approaches and why we used them. Below we provide further technical details for analyses related to the first two questions, as well as for preliminary data treatment.

Preliminary data treatment

For multivariate community analyses, all taxon abundance data were square root-transformed. This reduced the influence of the most abundant taxa and increased the influence of rare taxa in the analyses. Bray-Curtis coefficients were computed for each pair of samples (i.e., site-years) to measure assemblage similarity. Following recommendations [1], we added a "dummy" taxa with an abundance of 1 to every site-year in order to avoid cases where the Bray-Curtis coefficient is undefined when both samples in a pair lack organisms.

For univariate single-taxon analyses, biological data were transformed (specific transformation indicated in tables and figures) as needed to meet assumptions of normality and homogeneity of variance [2]. These assumptions were checked with residual plots which were also used to check for linearity in regression analyses.

Repeated measures analyses (Question 1, Table 1)

We used Permanova in the PRIMER7 software package to test multivariate community responses [3]. We generated multivariate means and plotted them as centroids for each time period and site group.

The resulting time trajectory of centroids through the four years of study (1 before and 3 after annual surveys) is analogous to a time trajectory of means; however, it conveys the magnitude and direction of community change but not the nature of change (i.e., how individual species changed). We used the pair-wise tests option in Permanova for simple main effects tests. We conducted separate Permanova analyses to test interaction contrasts for each site-group pair. We used lmer in the package lme4 in R [4] to test univariate responses and the phia package in R [5] for interaction contrasts and simple main effects tests.

Multiple regression analyses (Question 2, Table 1)

We conducted univariate linear multiple regressions to assess the response of a single taxon or summary statistic (e.g. total cover). We used lm in the package stats in R [6] for the dive data, and lme in the package nlme [7] for the video data because of the need to include (1) transect as a random effect and (2) a correlation structure to account for higher correlation of residuals between adjacent transect segments than between segments that were farther apart [2]. We used the auto-regressive model of order 1 (AR-1) correlation structure. We assessed predictor collinearity with variance inflation factors (VIFs), which indicate inflation of regression coefficient variance due to collinearity. VIFs of predictors in final models were $\leq$ 1.4.

We conducted multivariate linear multiple regressions assessing community response to predictors with distance based linear models (DISTLM) in Permanova [3]. We ran analyses for invertebrates and fish at our finest level of taxonomic resolution and also for taxa lumped to a coarser level and found results to be similar between the two. We used the shorter “coarse” taxa list to examine how response varied among taxa. Multivariate models were visualized with distance based redundancy analysis (dbRDA) plots [3]. The number of dbRDA axes is equal to the number of predictors in the model. All axes combined explain 100% of the fitted community variation (i.e. the variation explained by the model) and a

percentage of the total community variation (akin to an R^2 value). We plotted the first two axes against each other and report percent of fitted and total variation explained by each of those axes. We overlaid vectors representing predictor effects and taxon responses on the dbRDA plots. Predictor vectors indicated direction and strength of the multiple partial correlation between each predictor and each dbRDA axis. Multiple partial correlations are conditional on the other variables in the model and indicate the correlation between a variable and the axes when all other variables are held constant. Taxon vectors indicated the direction and strength of the raw (not conditional) correlation between each taxon and each axis. The arrangement of taxon and predictor vectors informed which taxa we used in our univariate analyses.

We conducted univariate nonlinear multiple regressions assessing the response of vegetation abundance to the predictors with general additive models (GAMs) [8]. The shape of the response-predictor relation is unrestricted in GAM; curves of any shape can be fit. GAMs for both dive and video data indicated a nonlinear relationship between vegetation abundance and reflectance change, and an interactive effect of reflectance change and initial depth on vegetation abundance. Exploratory GAMs assessing relations between invertebrate or fish responses and the predictors did not suggest nonlinearity or interactive effects. We therefore included a quadratic term for reflectance change and an interaction term for reflectance*depth in our linear (univariate and multivariate) multiple regression models for vegetation to account for the curved and interactive relationships suggested by GAM, but no such terms were included in the models for invertebrates or fish. We used GAMs only for assessing vegetation, but linear models for assessing all groups (vegetation, invertebrates, and fish), because GAMs provided the best description of response-predictor relationships for vegetation whereas linear models provided a means of comparing explained variance and the relative importance of different predictors across all groups.

We used the routine gam in mgcv in R for dive data GAMs, and gamm in mgcv for video data GAMs because of the need to include a random effect and correlation structure as described above for linear models [8]. In the context of GAMs, testing for an interaction between two continuous variables (e.g. reflectance change and depth) means testing the significance of a two-dimensional smooth. We used a tensor product smooth, because it is appropriate for predictors measured on different scales, and ti terms to specify it in the model because such terms are designed for a main effects-plus-interaction structure.

For all multiple regressions (univariate and multivariate linear and univariate non-linear), non-significant predictors ($P > 0.05$) were dropped using backwards model selection [2]. For linear multiple regressions, we report delta R^2 values for each predictor in the final models to indicate predictor effect size. Delta R^2 is the increase in R^2 obtained (i.e., the additional variation explained) when the predictor is added to a model already containing all other predictors. The delta R^2 values do not sum to R^2 because of the overlap among predictors in the percentage of variation explained.

References

1. Clarke K, Gorley R. PRIMER v7: User Manual/Tutorial. PRIMER-E, Plymouth, UK2015.
2. Zuur AF, Ieno EN, Walker NJ, Saveliev AA, Smith GM. Mixed effects models and extensions in ecology with R. New York: Springer; 2009.
3. Anderson MJ, Gorley RN, Clarke KR. PERMANOVA+ for PRIMER: Guide to Software and Statistical Methods. Plymouth, UK: PRIMER-E; 2008.
4. Bates D. lme4: Linear Mixed-Effects Models using 'Eigen' and S4. 1.1-13 ed: R; 2017.
5. Rosario-Martinez HD. phia: Post-Hoc Interaction Analysis. 0.2-1 ed: R; 2015.
6. Team RC. The R Stats Package. 3.5.0 ed: R; 2017.
7. Pinheiro J, Bates D. nlme: Linear and Nonlinear Mixed Effects Models. 3.1-131 ed: R; 2017.
8. Wood S. mgcv: Mixed GAM Computation Vehicle with GCV/AIC/REML Smoothness Estimation. 1.8-17 ed: R; 2017.
