# Supplementary material for: Increased sediment load during a large-scale dam removal changes nearshore subtidal communities
Source: PLoS One. 2017 Dec 8;12(12):e0187742. doi: 10.1371/journal.pone.0187742 (PMC5722376; doi:10.1371/journal.pone.0187742)
Supplement: S2 Text — (PDF) [file pone.0187742.s015.pdf]

S2 Text: Limitations of BACI, diver observations, and light reflectance

The repeated measures Before-After-Control-Impact (BACI) analyses did not detect a dam removal effect for invertebrates or fish, as indicated by the site-group*year interaction term not being significant for any of the response metrics associated with these taxonomic groups (Table 3, main paper). Yet, dam removal obviously did affect invertebrates and fish. A dam removal effect was not found primarily because the dam removal-related change most strongly affecting invertebrates and fish, persistent sediment deposition, was not adequately represented by our site-groups. The “impact” (i.e., Mouth) site-group (site-group locations shown in Fig 1, main paper) included sites where deposition did not occur as well as sites where it did. When the analysis was rerun with the Mouth site-group broken into two groups, sites with and without deposition, a significant dam removal effect (i.e., a significant site-group*year interaction) was found for the swath survey invertebrate assemblage. This highlights a potential drawback—difficulty with identifying ahead of time where the impact is going to occur—of the BACI approach.

Detectability and identification of organisms are important issues for surveys conducted using underwater observation [1]. We do not think the inverse relations of tubeworm and flatfish densities, and sessile invertebrate cover to algal cover resulted because detectability increased when algal cover decreased. Tubes of the largest tube worm *Eudistylia vancouveri* projected well above the substrate and were visible to divers even with heavy algal cover. We also confirmed *E. vancouveri* increases by reviewing dive site transect videos. We moved algal foliage aside to assess sessile invertebrate primary cover at UPC survey points. Tubeworms, flatfish, and sessile invertebrates were the only taxa found to increase when algal cover decreased but not the only taxa that would have been easier to see. We identified and counted bivalves using their siphons which were sometimes retracted into the substrate

(i.e., out of sight) depending on season and environmental conditions [2]. This likely increased the variability of our bivalve counts but probably not in a systematic way that would bias assessment of their response to dam removal or sediment increases. Lumping species into our “coarse” taxa groups essentially corrected any species misidentifications. The issue with the coarse taxa groups is that they included multiple species that may not have all responded similarly. However, results for invertebrate and fish assemblage responses were generally similar between the fine and coarse taxa groups, suggesting that we adequately characterized community response to dam removal.

Remotely sensed light reflectance is strongly correlated with near-surface suspended sediment concentration [3]. However, during our study, there were a relatively small number of clear days providing usable reflectance data (S3 Table). This was particularly true for periods of high suspended sediment entering the Strait of Juan de Fuca from the river, because high sediment discharges from the river were often associated with rainfall during cloudy days [4]. Representation of suspended sediment was inconsistent among years because of the small sample size of clear days. In contrast, reflectance was a good measure of differences in suspended sediment concentration among sites at the points in time when images were taken. Data were usable for at least 90% of the sites (including video transect segments) in 86% of the images providing usable data, and for at least 50% of the sites in all the images we used.

Several issues remain for better characterizing spatial and temporal variation in suspended sediment, including the effects of such variation on benthic organisms, particularly macroalgae. Hydrodynamic models may be able to capture variability better than remotely-sensed light reflectance because of their potential to integrate river inputs with effects of tidally-driven currents. Ephemeral deposition and scour probably played a role in algal cover decreases at our sites, although we did not measure this throughout the study. Comparing the timing of deposition and scouring events with algal life histories

would give us a better understanding of how these processes affect benthic communities. Finally, developing a relationship between suspended sediment concentration and light attenuation at our sites would be useful for identifying light thresholds for different species and life stages of macroalgae.

References

1. Dolloff A, Kershner J, Thurow R. Underwater observation. In: Murphy BR, Willis DW, editors. Fisheries Techniques. Second ed. Bethesda, Maryland: American Fisheries Society; 1996.
2. Goodwin CL. The effects of season on visual and photographic assessment of subtidal geoduck clam (Panope generosa Gould) populations. Veliger. 1977;20:155-8.
3. Hudson B, Overeem I, McGrath D, Syvitski J, Mikkelsen A, Hasholt B. MODIS observed increase in duration and spatial extent of sediment plumes in Greenland fjords. The Cryosphere. 2014;8(4):1161-76.
4. Magirl CS, Hilldale RC, Curran CA, Duda JJ, Straub TD, Domanski M, et al. Large-scale dam removal on the Elwha River, Washington, USA: Fluvial sediment load. Geomorphology. 2015;246:669-86. doi: http://dx.doi.org/10.1016/j.geomorph.2014.12.032.
